# Lamin B1 coordinates the assembly of the nuclear lamina

**DOI:** 10.1242/jcs.265156

**Published:** 2026-10-15

**Authors:** JoonYoung R. Kim, Paul H. Kim, Yiping Tu, James Carroll, Jenna P. Koerner, Stephen G. Young, Loren G. Fong

**Affiliations:** ^1^ Department of Medicine, University of California, Los Angeles, Los Angeles, CA 90095, USA; ^2^ Department of Human Genetics, University of California, Los Angeles, Los Angeles, CA 90095, USA

**Keywords:** Nuclear lamina, Lamins, Lamin knockout cells, Protein organization, Microscopy

## Abstract

The nuclear lamina provides essential mechanical stability to protect the genome; yet, how lamins target the nuclear rim and assemble into an organized meshwork remains unclear. To address this, we generated smooth muscle cells lacking all three lamin genes (*Lmna*, *Lmnb1* and *Lmnb2*; ‘LKO cells’) and expressed individual lamins at physiological levels. All lamins localized to the nuclear rim but with distinct requirements: lamin B1 and lamin B2 required farnesylation, whereas prelamin A did not. Lamin C and mature lamin A also localized efficiently, demonstrating that C-terminal processing is not required for A-type lamin targeting. Despite correct targeting, lamins differed in their ability to form organized meshworks. Lamin B1 alone generated a normal fibrillar network, whereas lamin A, lamin C and lamin B2 formed disorganized structures with large gaps. Co-expression of lamin A and lamin C did not rescue this defect. In contrast, co-expression with lamin B1 restored proper meshwork organization for all lamins and was essential for nuclear lamina assembly during cell division. These findings identify lamin B1 as the central organizer of nuclear lamina architecture.

## Teaser

**Summary:** Among the nuclear lamins, lamin B1 plays a central role in building a stable nuclear scaffold, which reshapes our understanding of nuclear lamina structure and its reassembly during cell division.

## INTRODUCTION

The nuclear lamina is a ∼10 to 20-nm-thick fibrillar meshwork beneath the inner nuclear membrane that provides structural support for the nucleus. Deficiencies in nuclear lamins result in misshapen nuclei, reduced integrity of the nuclear membranes, and DNA damage (Chen et al., 2018; Earle et al., 2020; Vargas et al., 2012). Aside from providing support for the cell nucleus, the nuclear lamina is involved in a wide range of functions in the nucleus, including chromatin organization, protein trafficking, gene regulation, mechanotransduction and nuclear positioning (Chang et al., 2019; Muchir and Worman, 2007; Osmanagic-Myers et al., 2015; Wong et al., 2022). Although cells deficient in nuclear lamins are viable and retain the capacity to proliferate (Chen et al., 2018; Jung et al., 2014) and differentiate (Kim et al., 2013), the inactivation of nuclear lamin genes is lethal at the organismal level (Coffinier et al., 2011; Kim et al., 2011; Sullivan et al., 1999; Vergnes et al., 2004). In mice, loss of *Lmnb1* or *Lmnb2* results in defective neuronal migration in the developing brain and perinatal lethality (Coffinier et al., 2011; Kim et al., 2011; Vergnes et al., 2004); *Lmna* deficiency causes muscular dystrophy, cardiomyopathy and early death (Sullivan et al., 1999). In humans, *LMNA* pathogenic variants cause a variety of diseases (‘laminopathies’), including partial lipodystrophy, peripheral neuropathy, muscular dystrophy, cardiomyopathy and progeroid syndromes (Shin and Worman, 2022). Pathogenic variants of evolutionarily conserved amino acids in lamin B1 and lamin B2 have been linked to microcephaly, and overexpression of lamin B1 causes autosomal dominant leukodystrophy (Padiath, 2019; Parry et al., 2021).

The nuclear lamina in somatic cells is composed of A-type lamins (lamin A and lamin C, alternatively spliced products of *LMNA*) and B-type lamins (lamin B1 and lamin B2, products of *LMNB1* and *LMNB2*, respectively) (Gerace et al., 1984; Rober et al., 1989; Stewart and Burke, 1987). Each nuclear lamin is a type V intermediate filament protein (Parry et al., 1986) with a conserved structure consisting of a central α-helical rod domain flanked by globular head and tail domains. Nuclear lamins assemble into a coiled-coil homodimer that polymerizes in a head-to-tail manner into linear strands approximately 3.5 nm in diameter; these strands then multimerize into higher-order filaments, which are the building blocks of the nuclear lamina (Heitlinger et al., 1991; Stuurman et al., 1996; Turgay and Medalia, 2017). Super-resolution microscopy has documented that individual nuclear lamins form distinct but overlapping networks at the nuclear rim (Nmezi et al., 2019).

Each nuclear lamin contains a nuclear localization signal that is required for entry into the nucleus (Loewinger and McKeon, 1988). Lamin B1 and lamin B2 have a C-terminal CaaX motif that triggers three sequential posttranslational processing steps: farnesylation of the cysteine (‘C’), endoproteolytic release of the last three amino acids (–aaX), and carboxyl methylation of the newly exposed farnesylcysteine (Maske et al., 2003; Worman et al., 2009). These modifications increase the hydrophobicity of the C-terminus and are thought to promote lamin B1 and lamin B2 interactions with the inner nuclear membrane, complementing the interaction with integral inner nuclear membrane proteins (Foisner and Gerace, 1993; Ye and Worman, 1994). Blocking lamin B1 farnesylation interferes with nuclear rim targeting, resulting in an accumulation of lamin B1 in the nucleoplasm (Nmezi et al., 2019). Farnesylation of lamin B2 is presumed to be important for its targeting to the nuclear rim, but those studies were based on pharmacological inhibition of farnesyltransferase (Adam et al., 2013).

Prelamin A contains a CaaX motif and undergoes the same three CaaX motif processing steps but, unlike the B-type lamins, it undergoes an additional endoproteolytic cleavage. A zinc metalloproteinase, ZMPSTE24, cleaves 15 C-terminal residues from prelamin A (including the farnesylcysteine methyl ester), thereby releasing mature lamin A (646 amino acids) (Young et al., 2005). The significance of this final processing step is unclear. Studies of ‘lamin A-only’ mice (*Lmna*^LAO/LAO^), in which the 646-residue lamin A is synthesized directly (bypassing all posttranslational processing steps) revealed that mature lamin A reaches the nuclear rim (Coffinier et al., 2010). In interpreting those findings, the authors proposed that the lamin A in *Lmna*^LAO/LAO^ mice could reach the nuclear rim by interacting with B-type lamins, but this proposal was not tested. Lamin C in ‘lamin C-only mice’ (*Lmna*^LCO/LCO^) also reaches the nuclear rim but, again, it was unclear whether lamin C targeting to the nuclear rim depended on interactions with B-type lamins (Fong et al., 2006).

Electron microscopy studies have provided further insights into the organization of the nuclear lamina (Gerace and Huber, 2012; Goldberg and Allen, 1992). Studies of *Xenopus* oocytes demonstrated that a B-type nuclear lamin (L_III_) forms a lattice-like structure (Aebi et al., 1986). More recently, cryo-electron tomography studies of mammalian cells, along with immunogold labeling of lamin A/C and lamin B1, have revealed an organized nuclear lamin meshwork (Turgay et al., 2017). Super-resolution microscopy has provided evidence that lamin B1 and the A-type lamins form distinct meshworks at the nuclear rim (Nmezi et al., 2019; Xie et al., 2016), each with small, regularly spaced openings (Kim et al., 2024; Shimi et al., 2015; Stiekema et al., 2021; Turgay et al., 2017; Turgay and Medalia, 2017; Xie et al., 2016).

Structural abnormalities in the nuclear lamin meshwork have deleterious consequences. For example, the loss of lamin B1 or the expression of progerin (a pathogenic variant of prelamin A in Hutchinson–Gilford progeria syndrome; Eriksson et al., 2003) results in large, irregular gaps in the nuclear lamina (Kim et al., 2024; Shimi et al., 2015) and loss of nuclear membrane integrity (Kim et al., 2024). The loss of nuclear compartmentalization can lead to genome instability and cell death (Maciejowski and Hatch, 2020).

Even though the physiological and pathophysiological importance of the nuclear lamins has been recognized for years, the mechanisms governing nuclear lamin targeting to the nuclear rim and assembly of the nuclear lamins into an organized meshwork are incompletely understood. In the past, investigators have attempted to address these issues by inactivating a single nuclear lamin gene (leaving the expression of the other nuclear lamin genes unperturbed). This approach makes it nearly impossible to assess the capacity of each individual nuclear lamin to reach the nuclear rim and form an organized meshwork. Another limitation of this approach is that gene-inactivation strategies can be accompanied by unanticipated problems. Mutations designed to create a null allele (e.g. deletion of one or more exons) can trigger aberrant splicing events that result in a truncated nuclear lamin (detectable at RNA and/or protein levels) (Jahn et al., 2012; Kim et al., 2023). Another approach for understanding nuclear lamins has been to examine cultured cells that have been transfected with a nuclear lamin expression vector, typically a vector driven by a viral promoter. These studies are also difficult to interpret, both because of the concurrent expression of endogenous nuclear lamin genes and because the properties of nuclear lamins are dependent on (and highly sensitive to) the levels of nuclear lamin expression (Guo et al., 2014; Shimi et al., 2015). It is difficult to draw conclusions about the properties of nuclear lamins unless they are expressed at physiological levels.

In the current study, our goal was to assess the capacity of each individual nuclear lamin to reach the nuclear rim and assemble into an organized meshwork. We began by creating smooth muscle cells (SMCs) in which all exons and the complete coding sequences of *Lmna*, *Lmnb1* and *Lmnb2* were deleted (eliminating any possibility of aberrant splicing and the production of mutant proteins). Once nuclear lamin-null SMCs were established, we expressed individual nuclear lamins at physiological levels. This approach made it possible to assess the capacity of each nuclear lamin to reach the nuclear rim and form an organized nuclear lamin meshwork. Our studies provided unexpected insights into the intrinsic properties of individual nuclear lamins and nuclear lamina assembly.

## RESULTS

### Generating SMCs lacking nuclear lamin proteins

The coding sequences of the nuclear lamin genes in mouse aortic SMCs were deleted sequentially (first *Lmna*, then *Lmnb2* and *Lmnb1*) by CRISPR/Cas9 gene editing (Fig. 1A). The locations of the guide RNAs to delete *Lmna*, *Lmnb2* and *Lmnb1* are indicated in Fig. S1A. Complete gene deletions were confirmed by PCR with flanking oligonucleotide primers (Fig. 1B–D) and DNA sequencing (Fig. S1B). Quantitative reverse-transcription PCR (qRT-PCR) studies confirmed the absence of *Lmna*, *Lmnb2* and *Lmnb1* transcripts (Fig. S1C) and western blotting analysis (Fig. S1D) confirmed the absence of nuclear lamin proteins. The nuclear lamin-null SMCs are referred to as lamin knockout (LKO) cells.

**Fig. 1.**
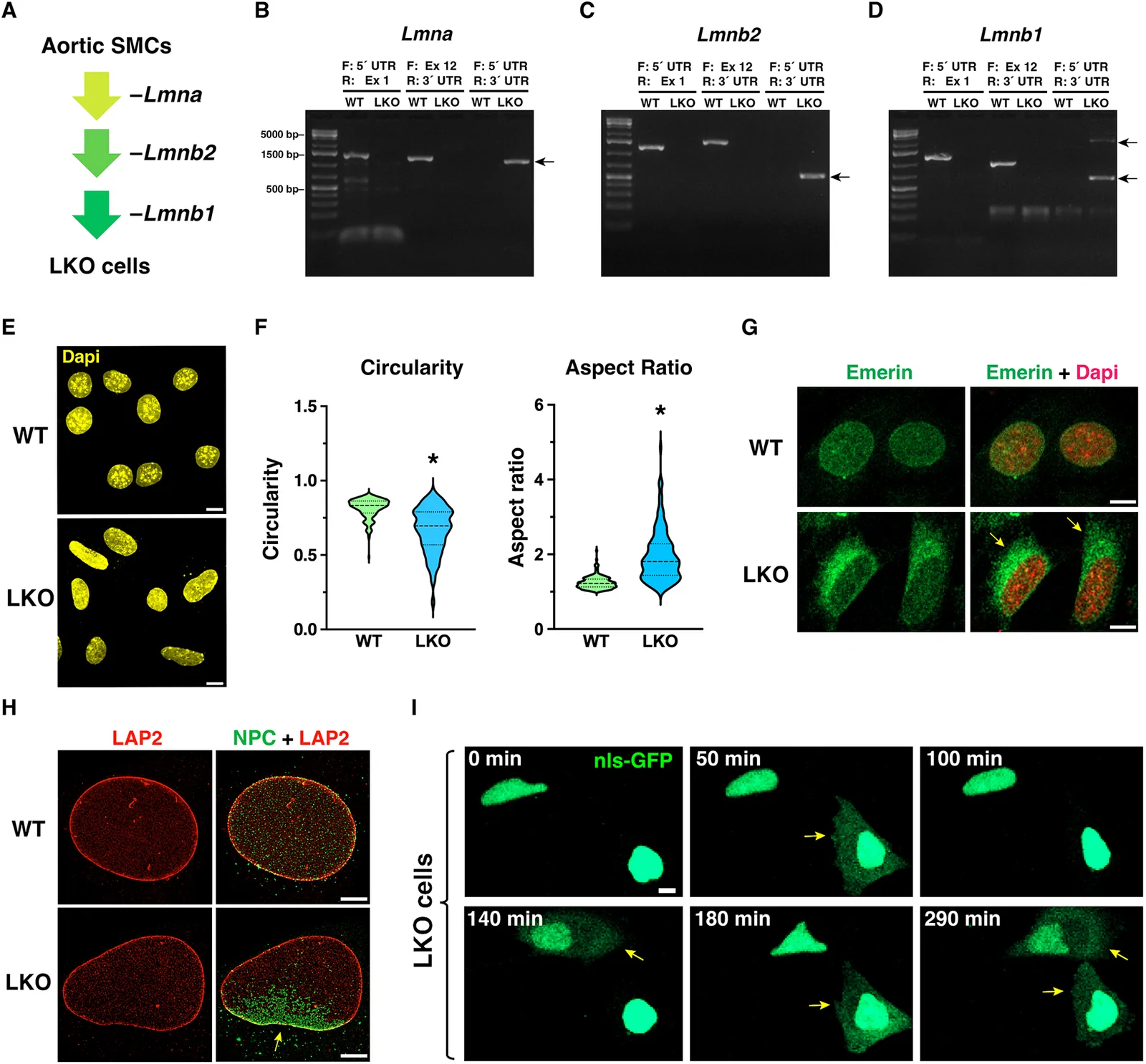
**Cells lacking *Lmna*, *Lmnb1* and *Lmnb2* are viable but exhibit abnormal nuclear shape, mislocalized nuclear pore complexes and emerin, and reduced integrity of the nuclear envelope.** (A) Diagram depicting the sequential deletion of *Lmna*, *Lmnb2* and *Lmnb1* in mouse aortic smooth muscle cells (SMCs), creating lamin knockout (LKO) cells. (B–D) PCR studies demonstrating the deletion of (B) *Lmna*, (C) *Lmnb2* and (D) *Lmnb1* in LKO cells. The locations of the forward (F) and reverse (R) primers are shown above each gel. The molecular size markers are shown on the left. The arrows point to amplicons produced in LKO cells with primers flanking each lamin gene. Two amplicons were produced for *Lmnb1*, which were verified by sequencing (Fig. S1B). Ex, exon; UTR, untranslated region. (E) Microscopy images of wild-type (WT) and LKO cells stained with DAPI (yellow). Scale bars: 10 μm. (F) Measurement of circularity and aspect ratio in WT (green; *n*=182) and LKO (blue; *n*=150) nuclei from three independent experiments. Circularity of 1.0 represents a perfect circle. Dashed lines represent the median; dotted lines represent the first and third quartiles. *P<0.0001 (Mann–Whitney test). (G) Microscopy images showing that emerin (green) is mislocalized in LKO cells (arrows). DNA was stained with DAPI (red). Scale bars: 10 μm. (H) Confocal microscopy images of WT and LKO cells stained with antibodies against nuclear pore complexes (NPC; green) and LAP2 (red). The arrow indicates the asymmetric distribution of NPCs. Scale bars: 5 μm. Low-magnification images are shown in Fig. S2. (I) Fluorescence microscopy images of live LKO cells expressing GFP with a nuclear localization signal sequence (nls–GFP; green). Images were collected every 10 min for ∼9 h (see Movie 1). Images were selected at different timepoints (shown in the upper left corner). Arrows point to cells with nuclear membrane ruptures, as judged by the presence of nls–GFP in the cytoplasm. Scale bar: 10 μm. Images in E,G–I represent a minimum of three independent experiments.

LKO cells exhibited irregularly shaped nuclei (Chen et al., 2018), as judged by circularity and aspect ratio (Svoren et al., 2023) (Fig. 1E,F), mislocalized emerin (EMD) (Fig. 1G) and asymmetric distribution of nuclear pore complexes (Chen et al., 2018; Guo and Zheng, 2015) (Fig. 1H; Fig. S2), but a normal distribution of LAP2 (encoded by *Tmpo*) at the nuclear rim (Fig. 1H), even though *Tmpo* expression was reduced (Fig. S1C). LKO cells also exhibited impaired nuclear membrane integrity (Chen et al., 2018), as judged by leakage of nuclear-targeted GFP into the cytoplasm (Denais et al., 2016) (Fig. 1I; Movie 1).

### Each nuclear lamin, when expressed in LKO cells, reaches the nuclear rim

To determine whether each nuclear lamin is independently capable of reaching the nuclear rim, we transiently transfected LKO cells with expression vectors for prelamin A (the precursor for lamin A), lamin C, lamin B1 or lamin B2. Cells that expressed nuclear lamins at physiological levels [i.e. levels observed in the parental wild-type (WT) SMCs] were analyzed 48 h post transfection by confocal fluorescence microscopy. Concurrently, we imaged each nuclear lamin protein in WT SMCs. As expected, the endogenous nuclear lamins in WT cells were all uniformly distributed at the nuclear rim (Fig. 2). In LKO cells, lamin B1 reached the nuclear rim and exhibited a uniform staining pattern (mirroring the pattern in WT cells). Lamin A, lamin C and lamin B2 also reached the nuclear rim, but the pattern of nuclear rim staining was irregular (discontinuous with multiple gaps).

**Fig. 2.**
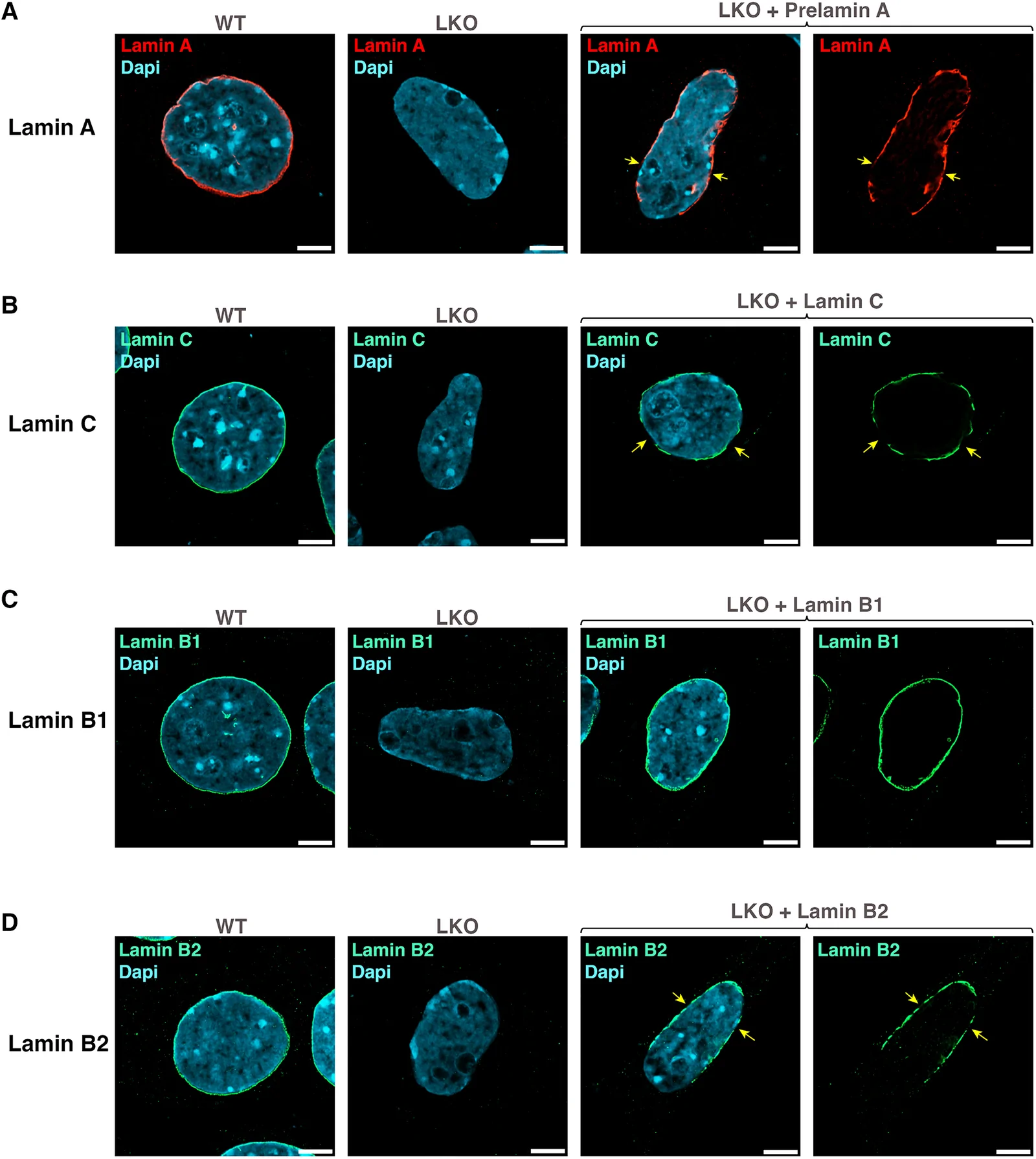
**A-type and B-type nuclear lamins traffic to the nuclear periphery independent of other nuclear lamins.** Individual nuclear lamins were transiently expressed in LKO cells and stained with directly labeled antibodies. DNA was stained with DAPI (blue). Cells with nuclear lamin expression levels equivalent to WT cells were examined by confocal fluorescence microscopy. Images were collected near the middle of nuclei. Representative images (from a minimum of three independent experiments) of WT cells, non-transfected LKO cells, and LKO cells expressing (A) prelamin A, (B) lamin C, (C) lamin B1 or (D) lamin B2 are shown. Note the gaps in nuclear lamin staining at the nuclear rim in LKO cells expressing lamin A, lamin C and lamin B2 (yellow arrows). Scale bars: 2 μm.

### Nonfarnesylated versions of lamin B1 and lamin B2 do not reach the nuclear rim

To assess the importance of the farnesyl lipid anchor for the targeting of lamin B1 and lamin B2 to the nuclear rim, we expressed mutant versions of these proteins harboring an amino acid substitution that eliminates protein farnesylation (a cysteine-to-serine substitution in the CaaX motif). In WT SMCs, lamin B1 and lamin B2 colocalized with an inner nuclear membrane protein (LAP2) at the nuclear rim (Fig. 3). Line scans across nuclei confirmed the targeting of lamin B1 and lamin B2 to the nuclear rim (Fig. 3B,D). When WT lamin B1 and lamin B2 were expressed in LKO cells, both proteins were seen at the nuclear rim (Fig. 3A–D). In contrast, nonfarnesylated versions of lamin B1 and lamin B2 did not reach the nuclear rim in LKO cells and were confined to the nucleoplasm (largely within inclusions) (Fig. 3A,C; Fig. S3). High-resolution imaging revealed that the nonfarnesylated lamin B1 in nucleoplasmic inclusions assembled into an ordered structure resembling a mesh (Fig. S3C,D). Electron micrographs revealed that the nonfarnesylated lamin B1 inclusions were composed of filaments surrounded by a space free of chromatin (Fig. S3E).

**Fig. 3.**
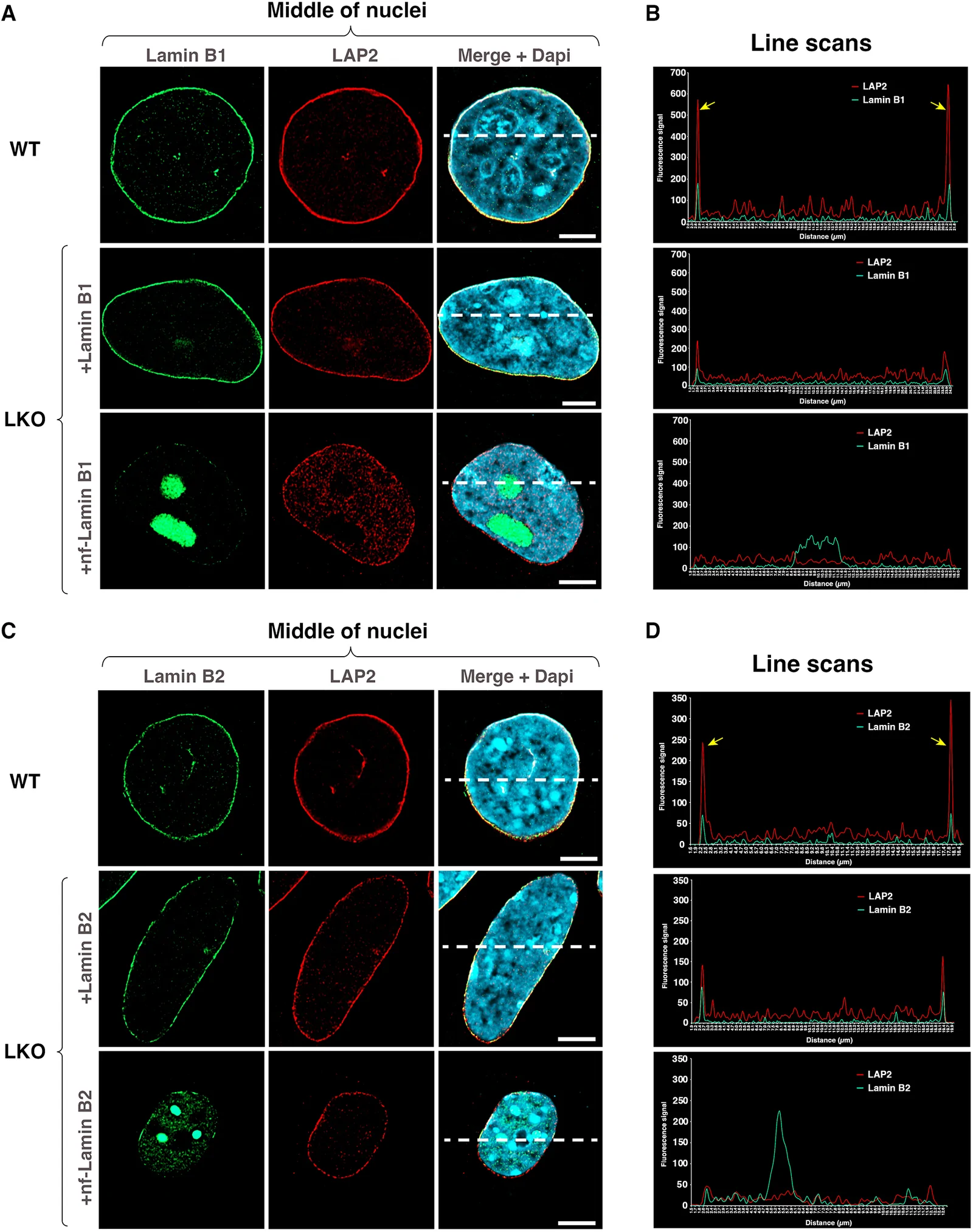
**Lamin B1 and lamin B2 depend on protein farnesylation to reach the nuclear rim.** LKO cells were transfected with plasmids for lamin B1, nonfarnesylated (nf)-lamin B1, lamin B2 and nf-lamin B2. WT cells were included as controls. After 48 h, cells were stained with antibodies against lamin B1, lamin B2 and LAP2. DNA was stained with DAPI (blue). LAP2 was used as a marker of the nuclear rim (red). Images were collected near the middle of nuclei. (A) Representative microscopy images (from three independent experiments) of WT cells and LKO cells transfected with lamin B1 (green) and nf-lamin B1 (green). Scale bars: 5 μm. (B) Line scans showing relative intensities of fluorescence signals for lamin B1 (green) and LAP2 (red) along the dashed lines in A. Arrows (yellow) point to the location of the nuclear rim. (C) Representative microscopy images (from three independent experiments) of WT cells and LKO cells transfected with lamin B2 (green) and nf-lamin B2 (green). Scale bars: 5 μm. (D) Line scans showing the relative intensities of fluorescence signals for lamin B2 (green) and LAP2 (red) along the dashed lines in C. Arrows (yellow) point to the location of the nuclear rim.

### Targeting of lamin A to the nuclear rim is not dependent on the posttranslational processing of prelamin A

The biogenesis of mature lamin A from prelamin A involves four processing steps: farnesylation of the cysteine (C) in the CaaX motif, removal of the –aaX, methylation of the farnesylcysteine and endoproteolytic release of 15 additional C-terminal residues (including the farnesylcysteine methyl ester) (Lutz et al., 1992). To examine the importance of these processing steps for nuclear rim targeting of lamin A, we transfected LKO cells with expression vectors for prelamin A, nonfarnesylated prelamin A (SSIM-prelamin A, containing a cysteine-to-serine substitution in the CaaX motif) (Holtz et al., 1989), or mature lamin A. The expression of each protein was confirmed by western blotting (Fig. S4). In WT cells, prelamin A was processed to mature lamin A, which, as expected, reached the nuclear rim and colocalized with LAP2 (Fig. 4A,B). In LKO cells, the lamin A derived from prelamin A processing also trafficked normally to the nuclear rim (Fig. 4A,B). We also observed robust nuclear rim targeting of nonfarnesylated prelamin A and directly synthesized mature lamin A (Fig. 4A,B), indicating that their nuclear rim targeting does not depend on prelamin A processing. Of note, however, the distribution of mature lamin A (from prelamin A), nonfarnesylated prelamin A and mature lamin A (produced directly) along the nuclear rim was not uniform but interrupted by multiple gaps (Fig. 4A). The distribution of LAP2 was also abnormal, closely mirroring the non-uniform distributions of the lamin A proteins. As noted previously (Fig. 1H), LAP2 in non-transfected LKO cells was evenly distributed at the nuclear rim (see also Fig. 4A).

**Fig. 4.**
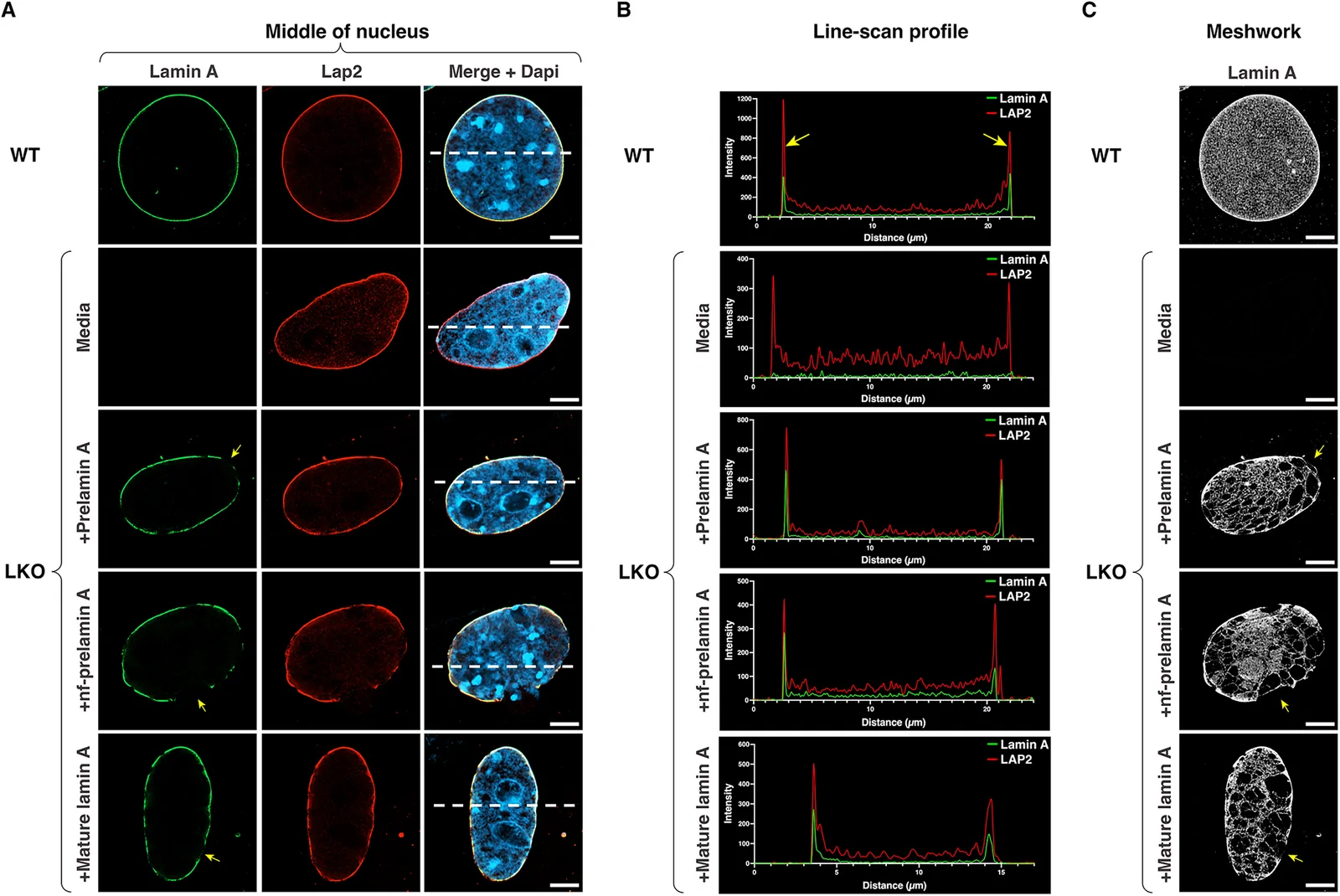
**The targeting of lamin A to the nuclear rim does not depend on the C-terminal posttranslational processing of prelamin A.** LKO cells were transfected with plasmids for prelamin A, nonfarnesylated prelamin A (nf-prelamin A) or mature lamin A. Cells incubated with cell culture medium alone were included as a negative control. After 48 h, the cells were stained with antibodies against lamin A (green) and LAP2 (red). DNA was stained with DAPI (blue). Images were collected near the middle of nuclei. (A) Representative images (from three independent experiments) of WT and transfected LKO cells. The yellow arrows point to gaps in lamin A staining at the nuclear rim. The dashed lines (white) in the merged images show where the line scans were performed. Scale bars: 5 μm. (B) Line scans showing the relative intensities of the fluorescence signals for lamin A (green) and LAP2 (red) along the dashed lines in A. The nuclear rim is identified by LAP2 staining at the nuclear periphery (arrows). (C) High-resolution confocal fluorescence microscopy images collected at the top of nuclei showing meshworks formed by lamin A in WT cells and three different lamin A proteins in transfected LKO cells. The arrows point to gaps in the protein meshworks (in C) that explain the gaps along the nuclear rim (in A). Scale bars: 5 μm.

To further characterize the abnormal distribution of A-type lamins at the nuclear rim, we performed high-resolution confocal imaging at the top of nuclei (allowing us to visualize nuclear lamin meshworks *en face*). In WT SMCs, the lamin A meshwork was regular with uniform small openings (Fig. 4C). In LKO cells, the meshworks formed by lamin A (from prelamin A processing), nonfarnesylated prelamin A and directly synthesized lamin A were irregular with large gaps and openings (Fig. 4C). The large gaps in the meshworks explain the discontinuous nuclear rim staining in the images recorded from the middle of the cell nucleus (see arrows in Fig. 4A,C).

### Investigating the nuclear lamin meshwork in LKO cells that co-express lamin A and lamin C

In WT cells, both lamin A and lamin C are uniformly distributed at the nuclear rim (Fig. 2). We initially suspected that co-expression of lamin A and lamin C in LKO cells might normalize meshwork morphology, but this was not the case. In LKO cells expressing both lamin A and lamin C, the meshwork remained irregular with large gaps (Fig. 5; Fig. S5). LAP2 staining was also irregular, mirroring the irregularities and gaps in the lamin A and lamin C meshworks (Fig. 5; Fig. S6).

**Fig. 5.**
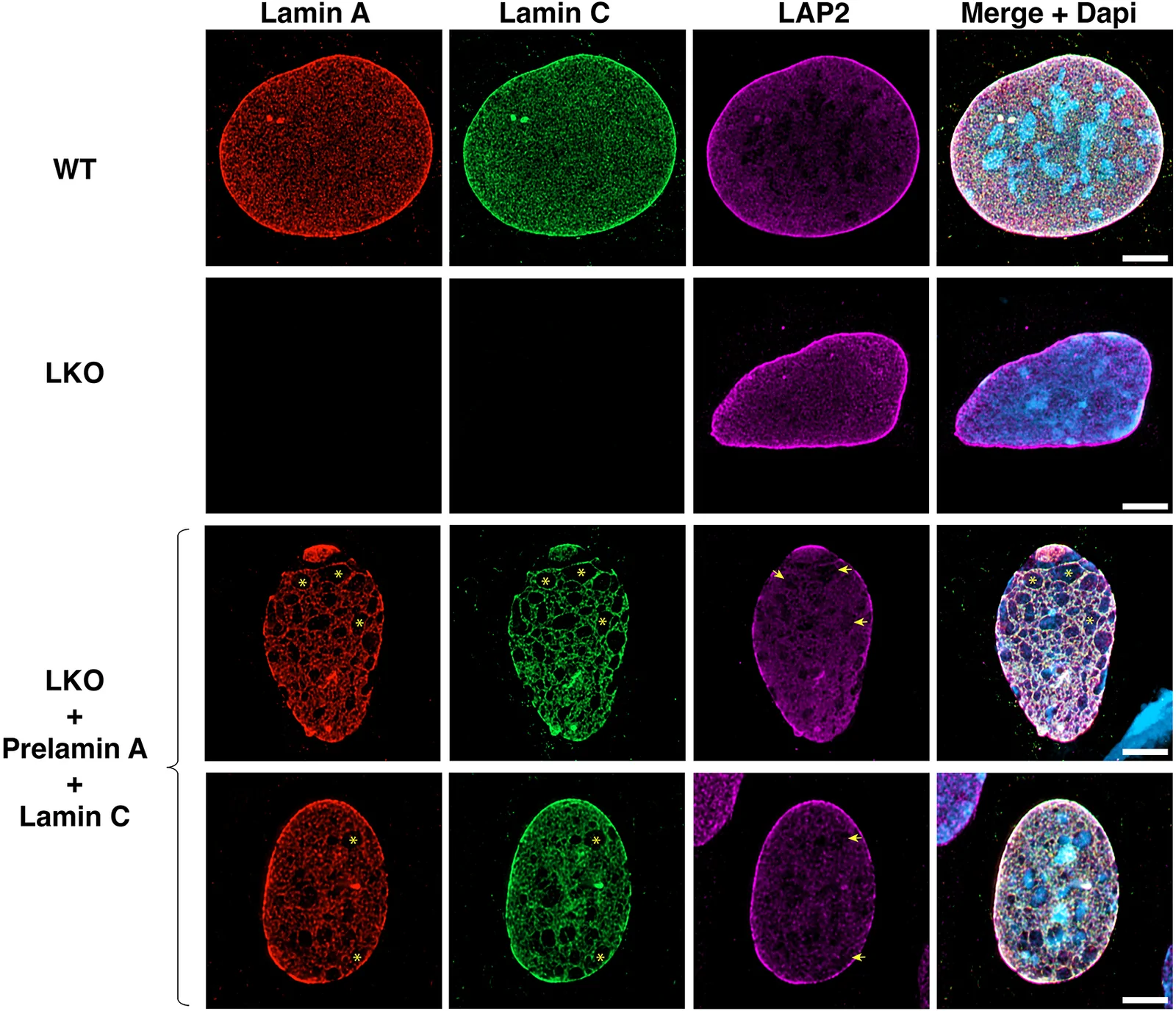
**Co-expression of lamin A and lamin C in LKO cells does not result in the formation of a normal meshwork.** LKO cells were transiently transfected with expression vectors for prelamin A and lamin C. After 48 h, the cells were stained with antibodies against lamin A (red), lamin C (green) and LAP2 (magenta). DNA was stained with DAPI (blue). Images were collected near the top of nuclei. Asterisks mark large holes in the lamin A and lamin C meshworks. Arrows point to gaps in the LAP2 structure that correspond to gaps in the lamin A and lamin C meshworks. Images represent three independent experiments. Scale bars: 5 μm.

The expression of physiological levels of lamin A in LKO cells resulted in an irregular meshwork with large gaps, but overexpression of lamin A (∼four times higher than its expression in WT SMCs) normalized meshwork morphology. Overexpression of lamin C failed to normalize the meshwork at the nuclear rim and resulted in the formation of lamin C aggregates (Fig. S7).

### Lamin C targeting to the nuclear rim of LKO cells requires its rod domain

To investigate sequences required for the targeting of A-type lamins to the nuclear rim, we generated mutant versions of lamin C in which its head and/or rod domains were replaced with the corresponding domains of lamin B1 (Fig. 6A). The expression of the lamin C chimeras in LKO cells was confirmed by western blotting (Fig. 6B). The lamin C chimera with the head domain of lamin B1 did not interfere with nuclear rim targeting (Fig. 6C), but the lamin C chimera with the rod domain of lamin B1 (and the chimera with both head and rod domains from lamin B1) abolished nuclear rim targeting and resulted in the accumulation of the chimeric proteins in the nucleoplasm (Fig. 6D; Fig. S8).

**Fig. 6.**
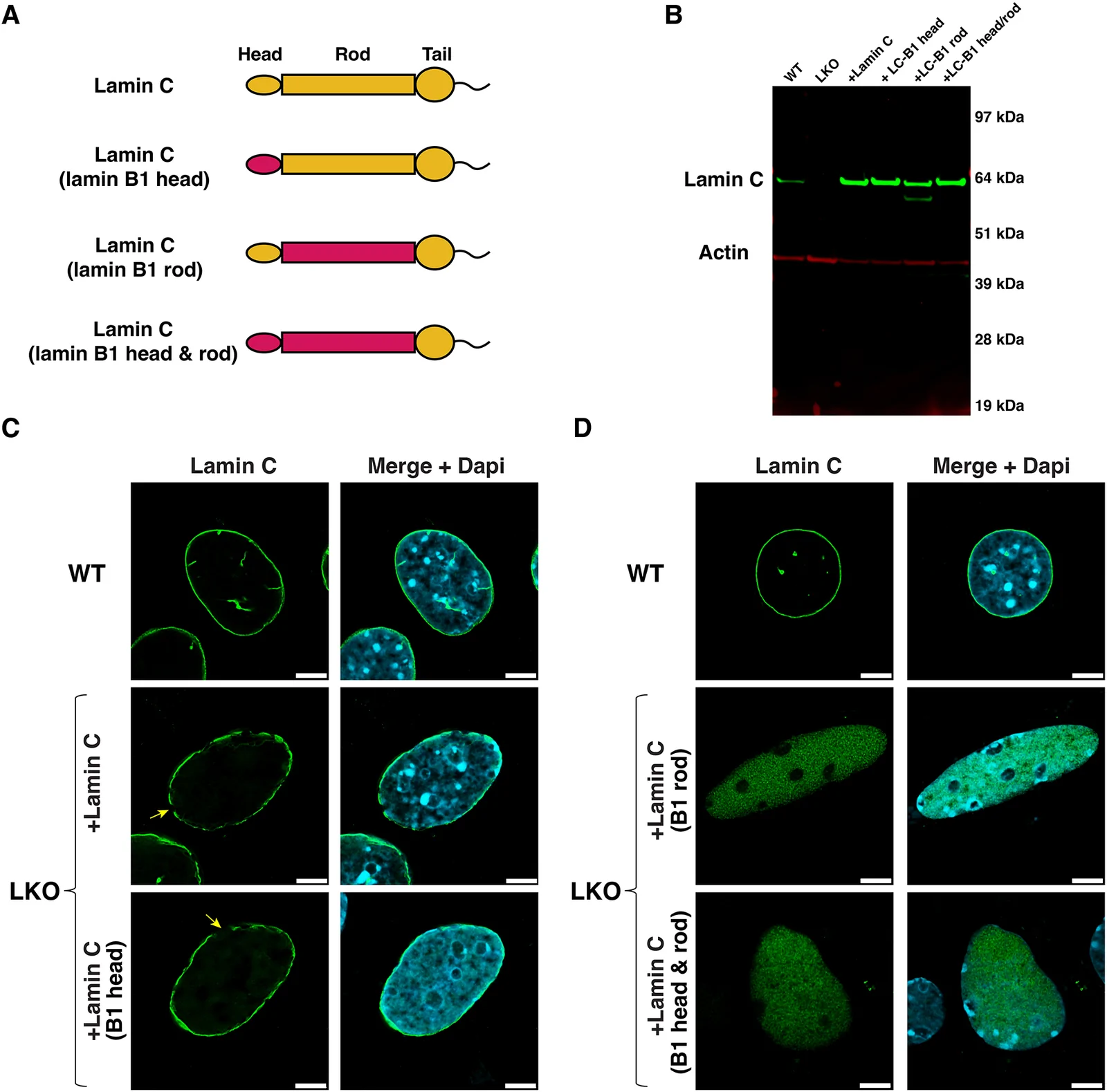
**Lamin C depends on rod domain sequences for nuclear rim targeting.** (A) The head and rod domains of lamin C (yellow) were replaced with the head and rod domains from lamin B1 (red) separately and together. (B) Western blot showing the detection of the mutant lamin C proteins using an antibody that binds to the C-terminal tail of lamin C. Molecular mass markers are shown on the right. Actin was measured as an internal control. (C,D) LKO cells were transiently transfected with WT and mutant forms of lamin C and stained with antibodies against lamin C (green). DNA was stained with DAPI (blue). WT cells were included as a control. Representative microscopy images (from three independent experiments) were collected near the middle of nuclei to assess the distribution of lamin C between the nuclear rim and nucleoplasm. Arrows point to gaps in the staining of lamin C at the nuclear rim. Scale bars: 5 μm.

### In LKO cells, lamin B1 is the only nuclear lamin that is capable of forming a morphologically normal meshwork

In WT SMCs, all of the nuclear lamins are distributed uniformly at the nuclear rim (Fig. 2) and form regular meshworks with small openings (Fig. 7A). When lamin A and lamin C were expressed independently in LKO cells, both formed irregular meshworks with large gaps. When lamin B2 was expressed in LKO cells, it was distributed asymmetrically (Fig. 7B; Fig. S9). However, when lamin B1 was expressed in LKO cells, the meshwork was regular with uniformly spaced small openings.

**Fig. 7.**
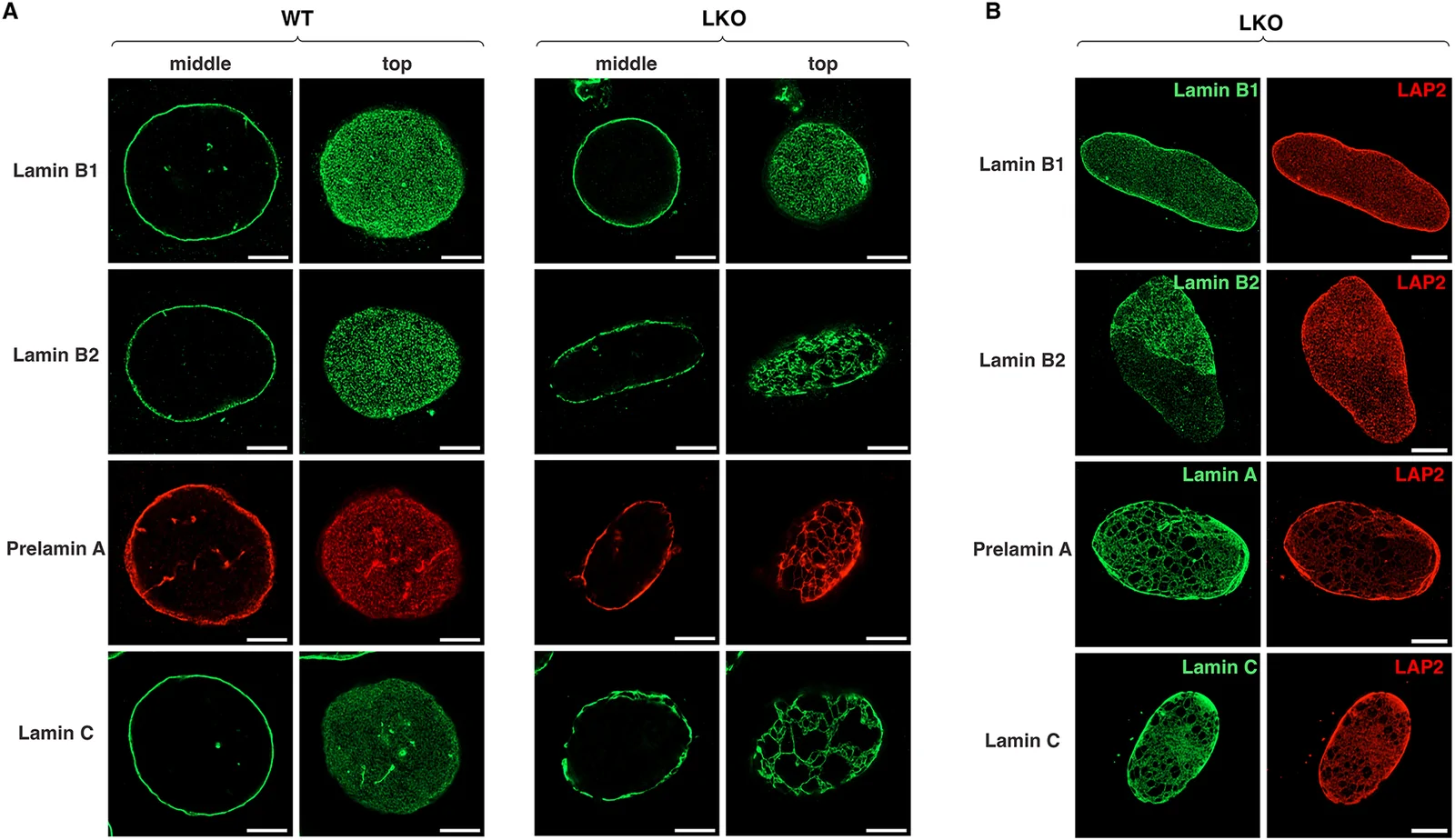
**Lamin B1 is the only nuclear lamin, when expressed alone in LKO cells, that forms a morphologically normal meshwork.** LKO cells were transiently transfected with expression plasmids for lamin B1, lamin B2, prelamin A and lamin C. After 48 h, the cells were stained with fluorescently labeled antibodies against lamin B1, lamin B2, lamin A and lamin C. Some cells were also stained with antibodies against LAP2. (A) Confocal microscopy images of lamin B1, lamin B2, prelamin A and lamin C in WT and transfected LKO cells. Images were captured near the middle and the top of the cell nucleus. Scale bars: 5 μm. (B) Images of LKO cells expressing lamin B1, lamin B2, prelamin A and lamin C and stained with antibodies against individual nuclear lamins (green) and LAP2 (red). Images in A,B represent three independent experiments. Scale bars: 5 μm.

### Lamin B1 expression in LKO cells does not normalize the shape of the cell nucleus

Because lamin B1 expression forms a uniform meshwork in LKO cells, we speculated that it might also normalize the irregular shape of the cell nucleus (Fig. 1E,F). To test this possibility, nuclear shape (circularity and aspect ratio) was quantified in LKO cells in which lamin B1 expression was under the control of a doxycycline-inducible promoter (lamin B1-pTripz-LKO cells). The results were clear: the expression of lamin B1 in LKO cells at physiological levels did not alter the shape of the cell nucleus (Fig. 8).

**Fig. 8.**
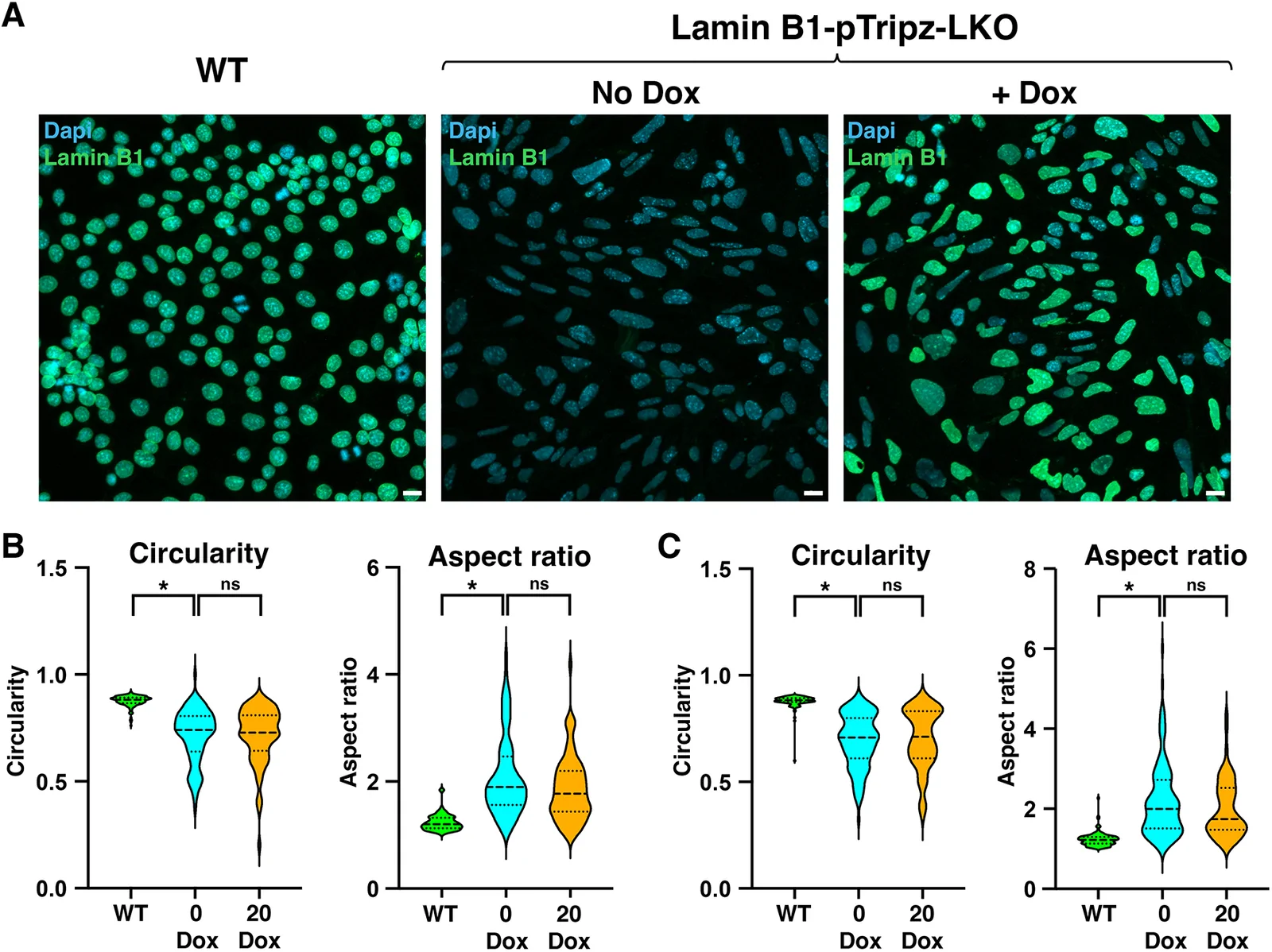
**Lamin B1 does not rescue the abnormal nuclear shape in LKO cells.** LKO cells expressing a doxycycline (Dox)-inducible construct for lamin B1 (Lamin B1-pTripz-LKO) were treated with 20 ng/ml doxycycline for 48 h to induce lamin B1 expression. WT cells and untreated lamin B1-pTripz-LKO cells were included as controls. (A) Representative images of WT cells as well as both untreated and doxycycline-treated lamin B1-pTripz-LKO cells that had been stained with antibodies against lamin B1 (green). DNA was stained with DAPI (blue). Scale bars: 20 μm. (B) Violin plots of nuclear circularity and aspect ratio in WT (*n*=148; green), LKO cells (*n*=92; blue) and LKO cells expressing lamin B1 (*n*=72; orange). (C) Violin plots of nuclear circularity and aspect ratio in a separate group of WT cells (*n*=119; green), LKO cells (*n*=115; blue), and LKO cells expressing lamin B1 (*n*=63; orange). Dashed lines in B,C represent the median; dotted lines represent the first and third quartiles. ns, not significant; *P<0.0001 (Kruskal–Wallis test with Dunn's multiple comparison post hoc test). Results in B,C represent findings from two independent studies.

### Lamin B1 expression normalizes the meshwork morphology in LKO cells expressing lamin A, lamin C or lamin B2

We suspected that lamin B1 might be sufficient to normalize the meshwork morphology in LKO cells that expressed prelamin A, lamin C or lamin B2. To test this idea, we transfected lamin B1-pTripz-LKO cells with expression vectors for prelamin A, lamin C and lamin B2. In the absence of doxycycline, the lamin A, lamin C and lamin B2 meshworks were irregular with large gaps. However, turning on lamin B1 expression with doxycycline normalized the meshwork morphology of all three nuclear lamins (Fig. 9; Fig. S10).

**Fig. 9.**
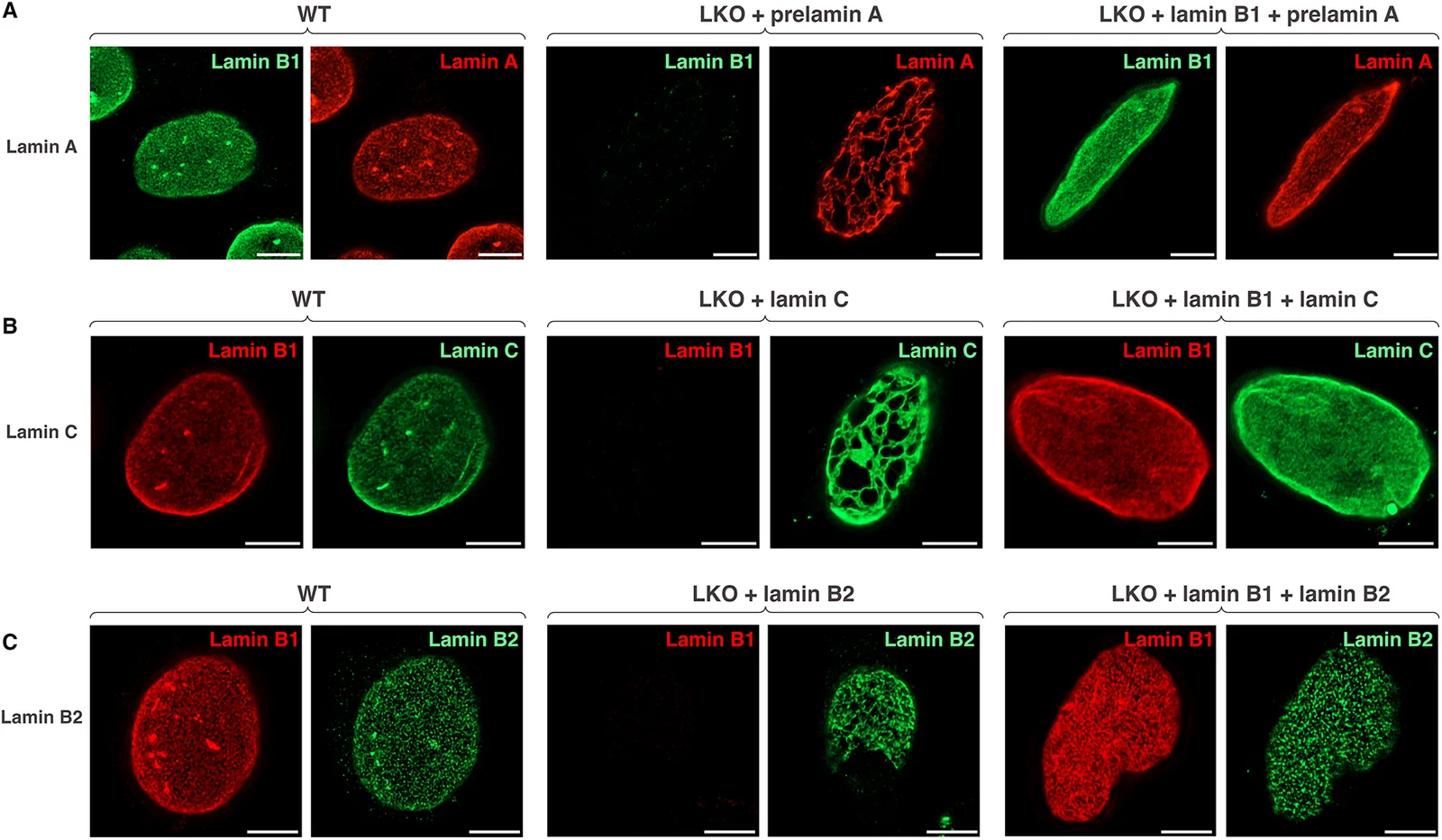
**The abnormal meshworks formed by lamin A, lamin C and lamin B2 are rescued by lamin B1.** Lamin A, lamin C and lamin B2 were transiently expressed in LKO cells that stably expressed a doxycycline-inducible lamin B1 construct. Lamin B1 was induced 4 h after cell transfection. After 48 h, the cells were stained with antibodies against lamin A, lamin C, lamin B2 and lamin B1. Images were recorded at the top of nuclei. Representative images (from three independent experiments) of WT cells and LKO cells expressing (A) lamin A, (B) lamin C or (C) lamin B2 in the presence or absence of lamin B1. Scale bars: 5 μm. Additional examples are shown in Fig. S10.

### Lamin C and lamin B2 form a morphologically normal meshwork in dividing cells in the presence of lamin B1

During cell division, the nuclear lamina disassembles and subsequently reforms in the two daughter cells (Picotto et al., 2025). To determine whether lamin B1 expression is essential for the assembly of a uniform nuclear lamina after cell division, we examined the impact of lamin B1 expression on lamin C and lamin B2 meshworks in synchronized dividing cells (with a double thymidine block–release protocol; Chen et al., 2025) (Fig. 10A). We examined both WT cells and lamin B1-pTripz-LKO cells that had been transfected with lamin C or lamin B2. In WT cells, the assembly of the nuclear lamina was observed 8 h after releasing the thymidine block, at which point lamin C and lamin B2 had formed uniform meshworks with small openings (Fig. 10B,C). In uninduced lamin B1-pTripz-LKO cells, the lamin C and lamin B2 meshworks were irregular with large gaps. However, after inducing lamin B1 expression with doxycycline, lamin C and lamin B2 formed uniform meshworks similar to those in WT cells.

**Fig. 10.**
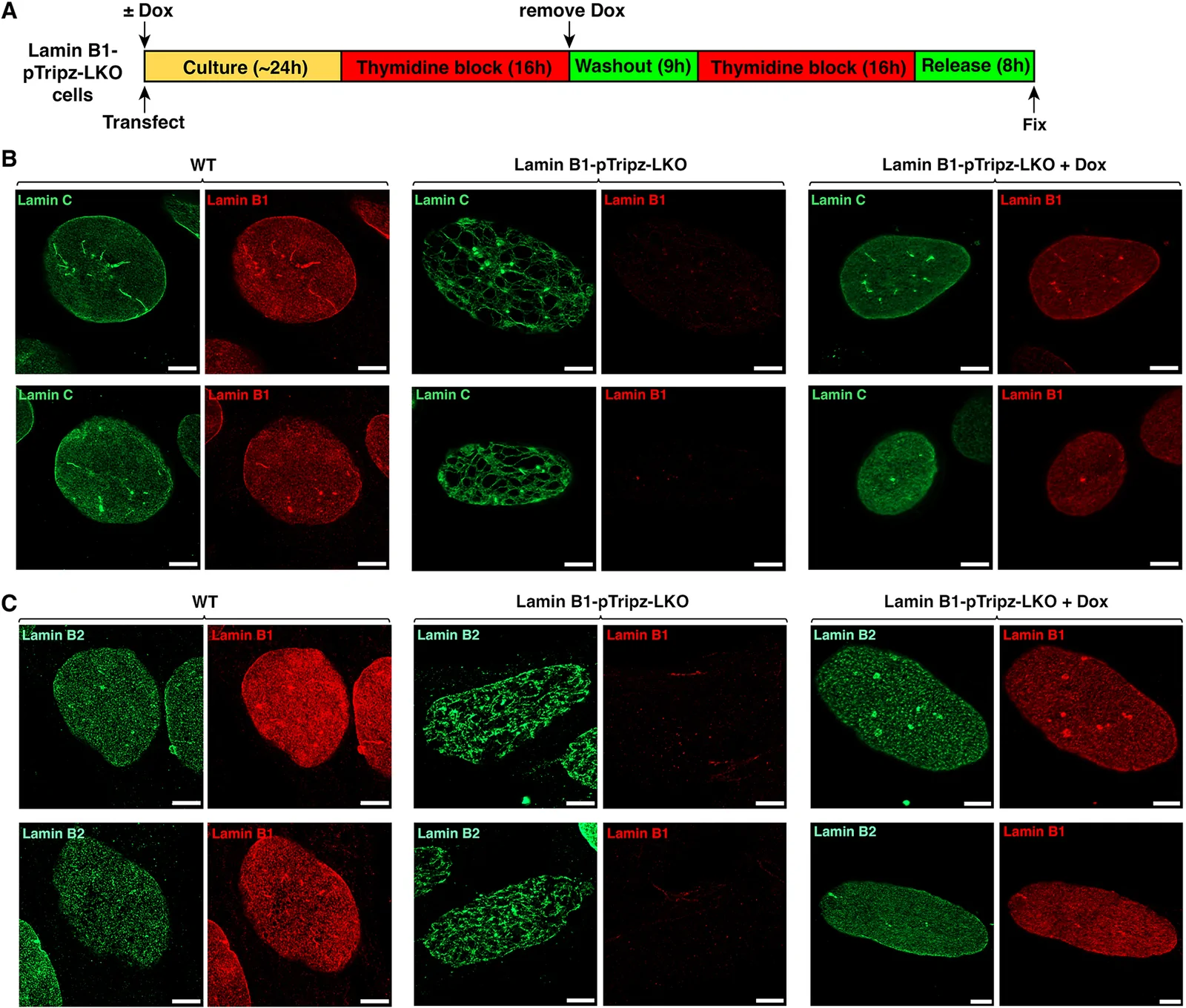
**Lamin C and lamin B2 form a morphologically normal meshwork in dividing cells in the presence of lamin B1 expression.** (A) Timeline to study lamin reassembly in synchronized dividing cells. Lamin B1-pTripz-LKO cells were transfected with lamin C or lamin B2. Lamin B1 synthesis was induced with doxycycline (Dox). WT cells and non-induced cells were included as controls. On the next day, cell division was paused with a double thymidine block (16 h thymidine, followed by 9 h washout, followed by 16 h thymidine). Cell division was resumed and, after 8 h, the cells were fixed and stained with antibodies. (B) Images of WT cells and LKO cells transfected with lamin C after staining with antibodies against lamin C (green) and lamin B1 (red). (C) Images of WT cells and LKO cells transfected with lamin B2 after staining with antibodies against lamin B2 (green) and lamin B1 (red). Images in B,C were collected at the top of nuclei and represent two independent experiments. Scale bars: 5 μm.

The organizing role of lamin B1 was also evident in *Lmnb1*^−/−^ SMCs. In these cells, the endogenous lamin C and lamin B2 proteins formed irregular meshworks characterized by multiple large gaps (Fig. S11).

## DISCUSSION

In the current study, we sought to understand targeting of individual nuclear lamins to the nuclear rim as well as their ability to form an organized nuclear lamin meshwork. To pursue this objective, we created nuclear lamin-null SMCs (LKO cells) in which the coding sequences of the three nuclear lamin genes were deleted. In these LKO cells, we expressed individual nuclear lamins at physiological levels (i.e. levels comparable to those in parental WT SMCs). This strategy made it possible to assess the properties of each nuclear lamin independent of its interactions with other nuclear lamin proteins. When expressed independently in LKO cells, lamin B1 and lamin B2 reached the nuclear rim, but nonfarnesylated versions of those proteins remained in the nucleoplasm, indicating that targeting of B-type lamins to the nuclear rim depends on protein farnesylation. Lamin C also reached the nuclear rim, which was unexpected because lamin C is not farnesylated and because its trafficking to the nuclear rim is thought to require the synthesis of prelamin A (Vaughan et al., 2001) or B-type lamins (Coffinier et al., 2010). Although lamin C targeting to the nuclear rim in LKO cells was initially surprising, it made sense in the context of our findings with nonfarnesylated prelamin A and mature lamin A. Nonfarnesylated prelamin A and mature lamin A, when expressed in LKO cells, also reached the nuclear rim, indicating that protein farnesylation (and the subsequent C-terminal processing steps) are not required for the targeting of A-type lamins to the nuclear rim.

To gain insights into the sequences required for nuclear rim targeting of A-type lamins, we expressed lamin C–lamin B1 chimeras in which the head and/or rod domains of lamin C were replaced by the corresponding sequences of lamin B1. A lamin C chimera with the head domain of lamin B1 reached the nuclear rim, whereas a lamin C chimera with the rod domain of lamin B1 did not. Thus, the rod domain of lamin C (which is shared by lamin A) appears to be required for nuclear rim targeting, presumably by interaction with the inner nuclear membrane or with an inner nuclear membrane protein. Consistent with these observations, pathogenic variants located within the rod domain of lamin A/C have been associated with increased amounts of lamin A/C in the nucleoplasm (Ghosh et al., 2022; Manju et al., 2006; Raharjo et al., 2001).

Although each nuclear lamin is capable of reaching the nuclear rim, the lamins differ in their ability to assemble into a uniform meshwork. Lamin A, lamin C and lamin B2 form irregular meshworks containing large gaps, indicating that nuclear rim targeting of nuclear lamins is not sufficient for the assembly of an organized network. In contrast, the expression of lamin B1 in LKO cells results in a regular meshwork with small gaps, closely resembling the meshwork in WT SMCs. The molecular basis for the unique ability of lamin B1 to form a regular meshwork is unclear. However, it is noteworthy that nonfarnesylated lamin B1 assembles into mesh-like structures within the nucleoplasm (Fig. S3). This observation suggests that lamin B1 can self-assemble into a meshwork and that the assembly is independent of lamin B1 anchoring to the inner nuclear membrane. Additional investigations of the nonfarnesylated lamin B1 inclusions, along with mutagenesis studies, could provide insights into the mechanisms required for the assembly of lamin filaments into higher-order meshworks.

When expressed in LKO cells, lamin A and lamin C formed disorganized meshworks with large gaps and openings. In the case of lamin A, increased levels of expression normalized meshwork architecture (Fig. S7). In contrast, overexpression of lamin C failed to rescue meshwork morphology and instead resulted in the formation of lamin C aggregates. These observations underscore the importance of examining cells that express physiological levels of nuclear lamins (given that the level of nuclear lamin expression can alter meshwork morphology). In our studies, we quantified nuclear lamin expression in individual cells and restricted our analyses to cells in which the level of nuclear lamin expression was comparable to levels in WT cells. Because increased levels of lamin A generated a morphologically normal meshwork, we suspected that co-expression of lamin A and lamin C might result in the formation of a normal lamin A/C meshwork, but this was not the case. Co-expression of lamin A and lamin C, each at physiological levels, did not normalize meshwork morphology. In contrast, lamin B1 expression normalized the lamin A and lamin C meshworks. Lamin B1 also normalized the distribution of lamin B2 along the nuclear rim. These findings imply that lamin B1 is the key organizer of nuclear lamina architecture and that the other nuclear lamins form organized meshworks by interacting with lamin B1.

A lamin-coordinating role of lamin B1 is supported by several lines of evidence. During embryonic development, B-type lamins are expressed before A-type lamins (Rober et al., 1989), consistent with their primacy in creating an organized nuclear lamina. Additionally, during cell division, lamin B1 associates with nuclear membranes before A-type lamins in the postmitotic assembly of the nuclear lamina (Moir et al., 2000). In support of that model, our analyses of synchronized dividing cells revealed that the assembly of lamin B2 and lamin C into an organized meshwork after mitosis depends on lamin B1 (Fig. 10). The ability of lamin B1 to promote a uniform meshwork supports a hierarchical model in which lamin B1 nucleates and/or stabilizes higher-order lamin filament assembly. Thus, lamin B1 is not merely a structural component of the nuclear lamina – it is an organizer of lamina architecture. Loss of the nuclear lamina-organizing function in lamin B1-deficient cells likely explains the increased frequency of nuclear membrane ruptures (Vargas et al., 2012) and blebs (Vergnes et al., 2004) in those cells and could contribute to the small size and perinatal lethality in *Lmnb1*^−/−^ mice (Chen et al., 2019; Kim et al., 2011; Vergnes et al., 2004).

In LKO cells lacking a nuclear lamina, nuclear morphology is highly irregular. This outcome was not unexpected as the nucleus in LKO cells is presumably more deformable due to the loss of heterochromatin anchoring at the nuclear periphery (resulting in increased chromatin mobility) and the absence of constraints on nuclear size that are imposed by the nuclear lamina (Dahl et al., 2008; Wintner et al., 2020; Zheng et al., 2018). Consequently, nuclear deformation is less restricted, leading to irregular nuclear shapes in response to cytoskeletal forces during cell movement (see Movie 1). However, we were surprised that lamin B1 expression failed to normalize or partially improve nuclear shape in LKO cells. Previous studies in nuclear lamin-deficient embryonic fibroblasts showed that lamin B1 and lamin A exhibit nearly identical steady-state stiffness (Wintner et al., 2020), a key determinant of nuclear shape over long timescales (minutes and longer). Based on these findings, we expected that the formation of a morphologically normal lamin B1 meshwork would improve nuclear shape in LKO cells; however, that was not the case. Instead, our results suggest that nuclear shape is governed more by the viscous properties of the nucleus, which are primarily controlled by lamin A and chromatin (Wintner et al., 2020).

In non-transfected LKO cells, membrane LAP2 (mainly LAP2β) is distributed uniformly at the nuclear rim. In lamin A-expressing or lamin C-expressing LKO cells, however, LAP2 distribution was irregular, mirroring the gaps and irregularities in lamin A and lamin C meshworks (Fig. 5; Fig. S6). The ability of lamin A and lamin C to regulate the distribution of LAP2 in LKO cells was unexpected because A-type lamins are thought to exert minimal effects on LAP2β distribution (Foisner and Gerace, 1993). Our findings imply that lamin A and lamin C interact, directly or indirectly, with LAP2β. In support of this proposal, we previously demonstrated that increased expression of LAP2β normalizes the grossly abnormal A-type lamin meshwork in SMCs that express progerin (Kim et al., 2024). We further propose that the interactions between A-type lamins and nuclear membrane proteins (Wilson and Foisner, 2010) could be important for the targeting of lamin A and lamin C to the nuclear rim in LKO cells.

The strategy that we used in the current study – first creating cells lacking the complete coding sequences of all three nuclear lamin genes and then expressing individual nuclear lamin proteins at physiological levels – was essential for elucidating the properties of individual nuclear lamins, in particular, their capacity to reach the nuclear rim and assemble into an organized meshwork. In earlier studies, a putative *Lmna* ‘null’ allele (Sullivan et al., 1999) (generated by removing several exons) was subsequently shown to produce an internally truncated lamin A (Jahn et al., 2012; Kim et al., 2023). Also, when we attempted to create nuclear lamin-null alleles in SMCs by deleting one more early exons in nuclear lamin genes, we often observed aberrant splicing events and residual expression of both transcripts and nuclear lamin proteins. For our current study, we were concerned that residual nuclear lamin production, even at low levels, could make it very difficult to interpret our findings and draw firm conclusions. Generating cells with complete gene deletions eliminated those concerns. In addition to generating nuclear lamin-null cells, our attention to expressing physiological levels of nuclear lamins was crucial because overexpression of nuclear lamins can influence both nuclear lamin distribution and organization (Guo et al., 2014; Shimi et al., 2015) (Fig. S7). We suspect that the findings that we observed in SMCs would be similar in other somatic cells, but that remains to be determined. In any case, the experimental methods used in our study will be broadly applicable to studying the function of nuclear lamins in mammalian cells.

In summary, we found that each nuclear lamin protein, when expressed independently in LKO cells, is capable of reaching the nuclear rim, but the nuclear lamins differ in their capacity to assemble into an organized meshwork. Lamin A, lamin C and lamin B2 reach the nuclear rim in LKO cells, but they cannot form a meshwork devoid of irregularities and gaps. Lamin B1 expression normalizes the abnormal meshworks formed by other nuclear lamins, indicating that lamin B1 plays an essential role in organizing the nuclear lamina.

## MATERIALS AND METHODS

### Cells

Immortalized mouse aortic SMCs (American Type Culture Collection, CRL-2797) were cultured in Dulbecco's modified Eagle medium (Invitrogen) supplemented with 10% (v/v) fetal bovine serum (HyClone), 1× nonessential amino acids, 2 mM glutamine, 1 mM sodium pyruvate and 0.2 mg/ml G418 at 37°C in the presence of 5% CO_2_. These cells are referred to as WT cells. LKO cells and LKO cells expressing a doxycycline-inducible lamin B1-pTripz construct were cultured under identical conditions.

### LKO cells

Mouse aortic SMCs lacking the complete coding sequences of *Lmna*, *Lmnb2* and *Lmnb1* were generated using CRISPR/Cas9-mediated non-homologous end joining (NHEJ) and homology-directed repair as described previously (Kim et al., 2024). Rather than generating small indels to induce frameshift mutations, we designed paired guide RNAs to excise the entire coding sequence of each lamin gene. This strategy was chosen to ensure complete gene disruption and eliminate the possibility of producing residual or truncated lamin proteins that could arise from alternative splicing and translation events. Guide RNAs were selected using the online Integrated DNA Technologies (IDT) CRISPR/Cas9 guide RNA design tool (https://www.idtdna.com/site/order/designtool/index/CRISPR_CUSTOM), with candidates chosen based on on-target and off-target scores greater than 90 (Table S2). *Lmna*-deficient SMCs were first generated using guide RNAs targeting sequences upstream of the *Lmna* 5′ end and downstream of the *Lmna* 3′ end. Guide RNAs and their complementary strands were ordered from IDT as single-stranded oligonucleotides, annealed to generate duplexes with compatible overhangs, and subcloned into the BbsI-linearized pX458-GFP vector (Addgene #48138). A Nucleofector II apparatus and the Cell Line T Nucleofector Kit (Lonza) were used to electroporate 3 μg of each vector into 2×10^6^ SMCs. After 48 h, cells with the top 1% GFP intensity were isolated by fluorescence-activated cell sorting and single cells were sorted into 96-well plates. After 10 days, genomic DNA was isolated from SMC clones using the DNeasy Kit (Qiagen) and analyzed by PCR with screening primers flanking the guide RNA cut sites. PCR products were sequence verified. Clones with distinct deletion junctions were selected to confirm deletion of both alleles. *Lmnb2*-deficient cells were then generated using the same strategy. Because the *Lmnb1* gene spans approximately 45.6 kb, deletion of the entire coding region by the same CRISPR/Cas9-mediated NHEJ knockout strategy was unsuccessful. Therefore, *Lmnb1*-deficient cells were generated using blasticidin- and zeocin-resistance knock-in cassettes, as described previously (Kim et al., 2024). Guide RNAs targeting sequences upstream of the *Lmnb1* 5′ end and within the *Lmnb1* 3′ untranslated region were subcloned into pX458-GFP. LoxP-flanked blasticidin- and zeocin-resistance cassettes containing homology arms were constructed to enable selection of cells with successful cassette integration into each allele. SMCs were electroporated with 3 μg of each CRISPR vector together with the corresponding donor cassette. After 48 h, cells were selected with 3 μg/ml blasticidin and 300 μg/ml zeocin for 14 days. Genomic DNA was extracted from resistant clones and screened by PCR. PCR products corresponding to the blasticidin- and zeocin-integrated alleles were sequenced to confirm targeted integration into each allele. A correctly targeted clone was expanded and treated with adenovirus expressing Cre recombinase to excise the integrated cassettes. The final LKO cell line, lacking the complete coding sequences of *Lmna*, *Lmnb2* and *Lmnb1*, was validated by genome sequencing, qRT-PCR, western blotting and immunofluorescence to confirm loss of detectable lamin transcripts and proteins.

### Generation of LKO cells harboring a doxycycline-inducible lamin B1 expression vector

LKO cells harboring a doxycycline -inducible pTripz expression vector for mouse lamin B1 (Lamin B1-pTripz) were generated as described previously (Kim et al., 2021). The expression vector was constructed by InFusion cloning (Takara Bio, Japan) of the lamin B1 cDNA into pTripz (Addgene #71307). The vector was verified by DNA sequencing. Packaging of lentivirus and transduction of cells were performed by the Vector Core at the University of California, Los Angeles (UCLA). Transduced cells were selected with 3 μg/ml puromycin for 2 weeks; individual clones were isolated by limiting dilution in 96-well plates. Clones were screened by western blotting and immunofluorescence staining. Lamin B1 expression was induced with doxycycline to achieve expression levels comparable to levels in WT cells.

### Plasmid constructs

Expression constructs for prelamin A, mature lamin A, lamin C, lamin B1 and lamin B2 were generated by InFusion cloning (Takara Bio, Japan) into the pCMV-XL5 mammalian expression vector (OriGene) linearized with EcoRI and XmaI. Prelamin A, mature lamin A, lamin C and lamin B2 coding sequences were amplified from mouse cDNA using primers containing 15-bp homology overhangs (Table S3). The mouse lamin B1 coding sequence was amplified from the commercial plasmid pCMV6-Lmnb1 (OriGene, MC219388). Nonfarnesylated forms of lamin B1 and prelamin A (SSIM-prelamin A) were generated using the QuikChange II XL Site-Directed Mutagenesis Kit (Agilent) according to the manufacturer's protocol by substituting the cysteine residue in the C-terminal CaaX motif with serine, as described previously (Kim et al., 2021). Substitution of the cysteine in the C-terminal CaaX motif of lamin B2 was performed by InFusion cloning with a single mutagenic primer. Primers used for mutagenesis are listed in Table S4. Lamin C chimeric constructs (Fig. 6A**)** were generated by replacing the head domain and/or rod domain of lamin C with the corresponding domains from lamin B1. Domain boundaries were assigned according to annotations in UniProtKB/Swiss-Prot entries P14733 for mouse lamin B1 (*Lmnb1*) and P48678 for mouse lamin C (*Lmna*). Chimeric lamin C constructs corresponding to the chimeric protein sequences in Table S5 were synthesized as double-stranded gene fragments by IDT and subcloned into a pCMV expression vector (OriGene) using InFusion cloning. All expression constructs were verified by sequencing prior to use.

### qRT-PCR

Total RNA was extracted with the RNeasy kit (Qiagen) and treated with DNase I (Ambion) according to the manufacturer's recommendation. RNA was reverse transcribed with random primers with SuperScript III cDNA Synthesis Kit (Invitrogen). cDNA samples were diluted in nuclease-free water and stored at −80°C. qRT-PCR was performed on a QuantStudio5 system (Thermo Fisher Scientific) with SYBR Green PCR Master Mix (Bioland). Transcript levels were calculated by the comparative cycle threshold method (Kim et al., 2018) and normalized to cyclophilin A expression. All primers used in the experiments are listed in Table S6.

### Transfection and expression analysis

LKO cells seeded in multi-chamber glass-bottom slides (ibidi) were transiently transfected with XFect (Takara) according to the manufacturer's recommendations. Cells were analyzed 48 h post transfection unless stated otherwise. Non-transfected (mock) LKO cells and WT cells were included as controls.

### Cell synchronization

WT SMCs or lamin B1-pTripz-LKO cells transfected with expression plasmids encoding lamin C or lamin B2 were incubated with 2 mM thymidine (Sigma-Aldrich) for 16 h (first block). The following day, cells were washed with normal culture medium and incubated in complete medium for 9 h (washout). Cells were then treated again with 2 mM thymidine for 16 h (second block) to arrest them at the late G1/early S-phase boundary (Chen et al., 2025). After release from the second block, cells were incubated in fresh medium for an additional 8–12 h before fixation.

### Immunofluorescence microscopy of the nuclear lamina

Cells were examined by confocal fluorescence microscopy as described previously (Kim et al., 2024). Fixed cells were permeabilized with 0.3% Triton X-100 and blocked at room temperature for 1 h. Cells were incubated with directly labeled fluorescent antibodies in blocking buffer at 4°C overnight, washed, and then post-fixed with paraformaldehyde. Nuclei were stained with DAPI, and images were recorded on a Zeiss LSM980 confocal microscope with Airyscan2 in super-resolution imaging mode with a scan speed of 5 and a Plan-Apochromat 63×/1.4 NA oil-immersion objective. Optical sections in the *z*-plane were recorded at a thickness of 0.15 μm. Images were processed with Airyscan Joint Deconvolution (Zeiss) with the following settings: sample structure, standard; maximum iterations, 10; and quality threshold, 0.00. For some samples, cells were co-stained with unlabeled antibodies against LAP2 (recognizing multiple LAP2 isoforms), LAP2β, emerin or nuclear pore complexes. Antibody details are listed in Table S1. Unless otherwise indicated, LAP2 was detected with the anti-LAP2 antibody that binds to multiple LAP2 isoforms.

Because expression levels affect nuclear lamin organization, only cells expressing WT lamin levels were selected for examination. WT levels were defined as the level expressed in WT SMCs. The amounts of antibody fluorescence were quantified in both WT and transfected LKO cells (stained simultaneously and under identical conditions) using the Zen Blue 3.5 Image Analysis Module (Zeiss). Transfected LKO cells expressing lamins at WT levels were selected for high-resolution imaging. To compare the distribution of nuclear lamins between the nuclear rim and nucleoplasm, single optical sections near the equatorial plane of nuclei were selected for analysis. To visualize and analyze the lamin meshworks at the nuclear periphery, maximum-intensity projections were generated from optical sections collected near the top of nuclei. Meshwork structure was assessed based on distribution, uniformity and size of openings. Representative images from 12–17 cells expressing WT levels are shown.

### Nuclear morphology and integrity assays

Nuclear morphology was assessed from fluorescence images of cells stained with DAPI. Images were imported into ImageJ for nuclear circularity and aspect ratio measurements. Cells undergoing cell division were excluded. Nuclear envelope integrity was evaluated by monitoring leakage of nucleoplasmic contents into the cytoplasm by live-cell imaging as described previously (Kim et al., 2021). Briefly, cells stably expressing GFP with a nuclear localization signal (nls–GFP) were seeded into two-well chamber slides with glass coverslip bottoms (Thermo Fisher Scientific) and cultured in complete culture medium. Mitomycin C (1 μg/ml) was added to the medium to trigger cell cycle arrest. The inhibition of cell proliferation facilitated quantification of nuclear membrane ruptures by eliminating mitotic cells. The cell culture chamber slide was mounted into a CO_2_- and temperature-controlled stage on a Zeiss LSM800 confocal laser-scanning microscope controlled by Zen Blue 2.3 software (Zeiss). The cells were visualized for 24 h at 37°C and 5% CO_2_ with a Plan Apochromat 20×/0.8 NA objective. Images from randomly selected fields of view were captured every 10 min.

### Western blotting

Lamin proteins were analyzed by western blotting as described previously (Kim et al., 2018). Urea-soluble protein extracts were size-fractionated on 4–12% gradient polyacrylamide Bis-Tris gels (Invitrogen) and transferred to nitrocellulose membranes. Protein expression was analyzed by western blotting with nuclear lamin-specific antibodies. Antibodies against actin or tubulin were included as a loading control. The antibodies and concentrations are listed in Table S1. The full uncropped blots are shown in Fig. S12.

### Electron microscopy

Cells were grown to about 90% confluency in six-well plates and fixed overnight in 2% glutaraldehyde and 1% paraformaldehyde in 0.1 M sodium cacodylate (pH 7.4). Following fixation, samples were washed three times in 0.1 M sodium cacodylate buffer (pH 7.4) and post-fixed in 2% osmium tetroxide and 1.5% potassium ferricyanide prepared in 0.1 M sodium cacodylate buffer (pH 7.4) for 30 min at room temperature. Samples were then washed three times in distilled water and stained with 2% uranyl acetate for 45 min at room temperature, followed by three additional washes with distilled water. Dehydration was carried out through a graded ethanol series (50%, 75%, 85% for 5 min each, then 100% for 10 min, two changes). Samples were infiltrated with a 2:1 mixture of resin to ethanol for 1 h, then transferred to 100% resin and incubated overnight. The resin was prepared by combining 25.7 g LX-112 (Ladd Research), 16 g nadic methyl anhydride (Polysciences), 8.3 g dodecenyl succinic anhydride (Polysciences) and 1 ml of benzyldimethylamine (EM Science) as accelerator. For embedding, BEEM capsules (Ted Pella) were filled with freshly prepared resin and inverted onto the cell monolayer, taking care to press each capsule firmly against the well bottom without lateral displacement. Resin was polymerized at 60°C for 48 h. After polymerization, the plates with BEEM capsules were heated on a hot plate and ‘popped off’ manually. Ultrathin sections (∼70 nm) were cut with a diamond knife on a Leica ARTOS 3D ultramicrotome and collected onto copper grids. Sections were examined on the Talos 120C transmission electron microscope (Thermo Fisher Scientific) operated at 120 kV. Images were recorded with the 4k×4k Ceta CMOS camera (Thermo Fisher Scientific).

### Statistical analysis

Quantitative data, including nuclear shape measurements, were analyzed with standard statistical methods. Differences between groups were evaluated with a Mann–Whitney test or a Kruskal–Wallis test with Dunn's post hoc comparison analyses.

## Supplementary Material



10.1242/joces.265156_sup1 Supplementary information
