# Supplementary material for: Lamin B1 coordinates the assembly of the nuclear lamina
Source: J Cell Sci. 2026 Oct 15;139(19):jcs265156. doi: 10.1242/jcs.265156 (PMC13741955; doi:10.1242/jcs.265156)
Supplement: Supplementary file 1 [file joces-139-265156-s1.pdf]

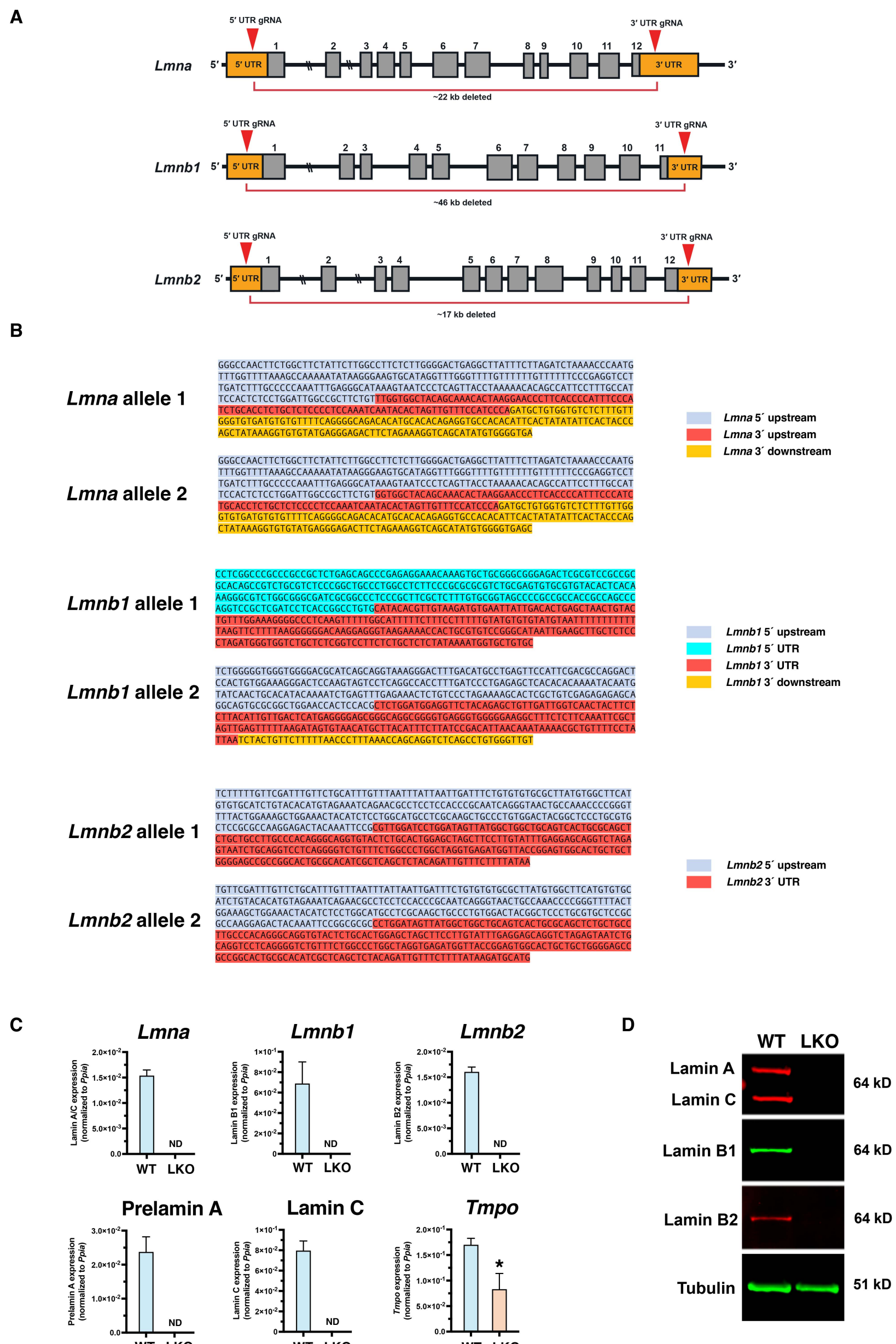

Fig. S1. Absent *Lmna*, *Lmnb1*, and *Lmnb2* expression in LKO cells. (A) The locations of the guide RNAs to delete *Lmna*, *Lmnb1*, and *Lmnb2* are indicated with red arrowheads. (B) Genomic sequencing demonstrates complete deletion of *Lmna*, *Lmnb2*, and *Lmnb1* coding sequences in LKO cells. Sequencing primers were located in the 5′ and 3′ UTRs or in sequences upstream and downstream from the 5′ and 3′ UTRs. Sequencing demonstrated that each allele of *Lmna*, *Lmnb2*, and *Lmnb1* had a different mutation, but all coding sequences were deleted in both alleles. Sequences are color coded (see legend). (C) Quantitative RT-PCR of RNA from WT and LKO cells shows that *Lmna*, *Lmnb2*, and *Lmnb1* transcripts are absent in LKO cells. ND not detected. Two-tailed Student's t-test. **P* < 0.01. (D) Western blot comparing the expression of lamin A, lamin C, lamin B1, and lamin B2 in WT and LKO cells. Tubulin was assessed as an internal control. Molecular weight markers are shown on the right.

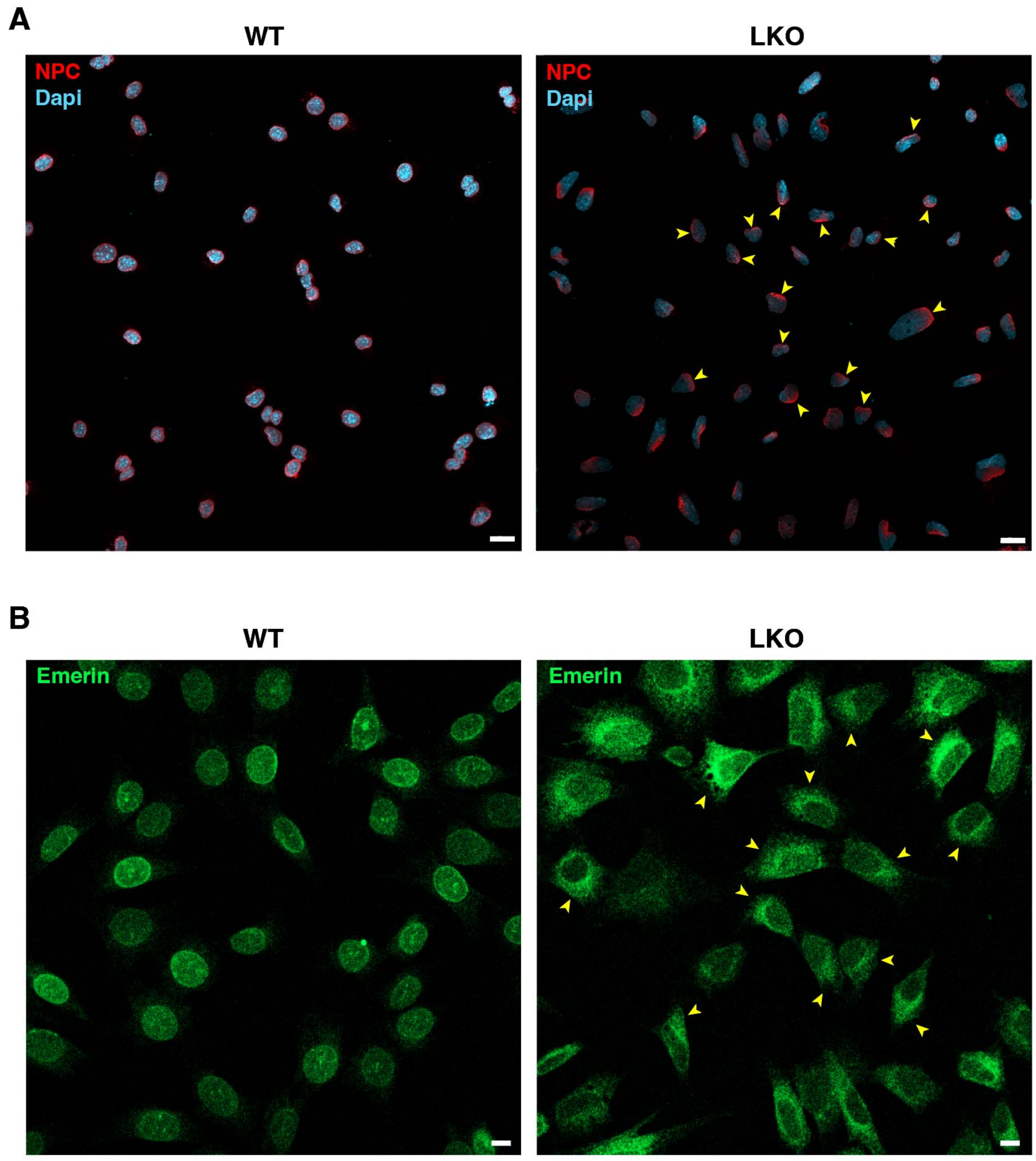

Fig. S2. The distribution of nuclear pore complexes and emerin in LKO cells is abnormal. (A) Representative low magnification microscopy images of WT and LKO cells stained with antibodies against nuclear pore complexes (NPC; *red*). DNA was stained with Dapi (*blue*). Scale bar, 20 µm. Arrowheads (*yellow*) point to examples of nuclei with polarized NPCs. (B) Representative low magnification microscopy images of WT and LKO cells stained with antibodies against emerin (*green*). Scale bar, 10 µm. Arrowheads (*yellow*) point to examples of cells with mislocalized emerin.

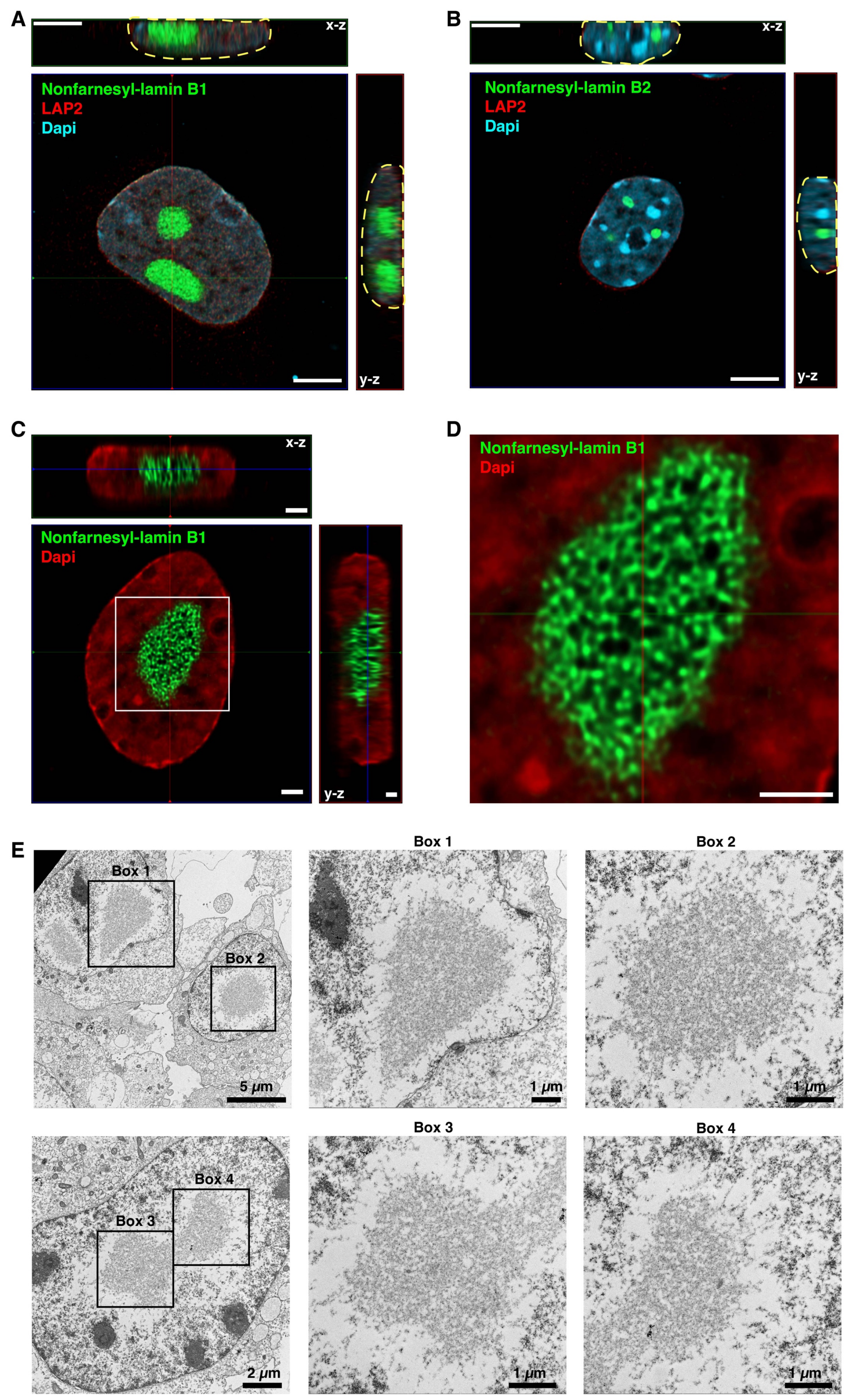

Fig. S3. Nonfarnesylated lamin B1 and nonfarnesylated lamin B2 form inclusions inside nuclei. Orthogonal images of nuclei in LKO cells expressing (A) nonfarnesyl-lamin B1 (*green*) or (B) nonfarnesyl-lamin B2 (*green*). DNA was stained with Dapi (*blue*). The same nuclei are shown in Figures 3A and 3C. The boundaries of nuclei in the *x–z* (top) and *y–z* planes (side) are marked with a dotted yellow line. Scale bar, 5 µm. (C) Orthogonal images of a nucleus with a nonfarnesyl-lamin B1 (*green*) inclusion. DNA was stained with Dapi (*red*). Scale bar, 2 µm. Scale bar in *y-z* plane, 1 µm. (D) Enlarged view of the boxed area in panel C. Scale bar, 2 µm. (E) Electron micrographs of LKO cells with nonfarnesyl-lamin B1 inclusions inside nuclei. Enlarged views of the boxed areas (1–4) are shown on the right. Scale bars are defined in the figures.

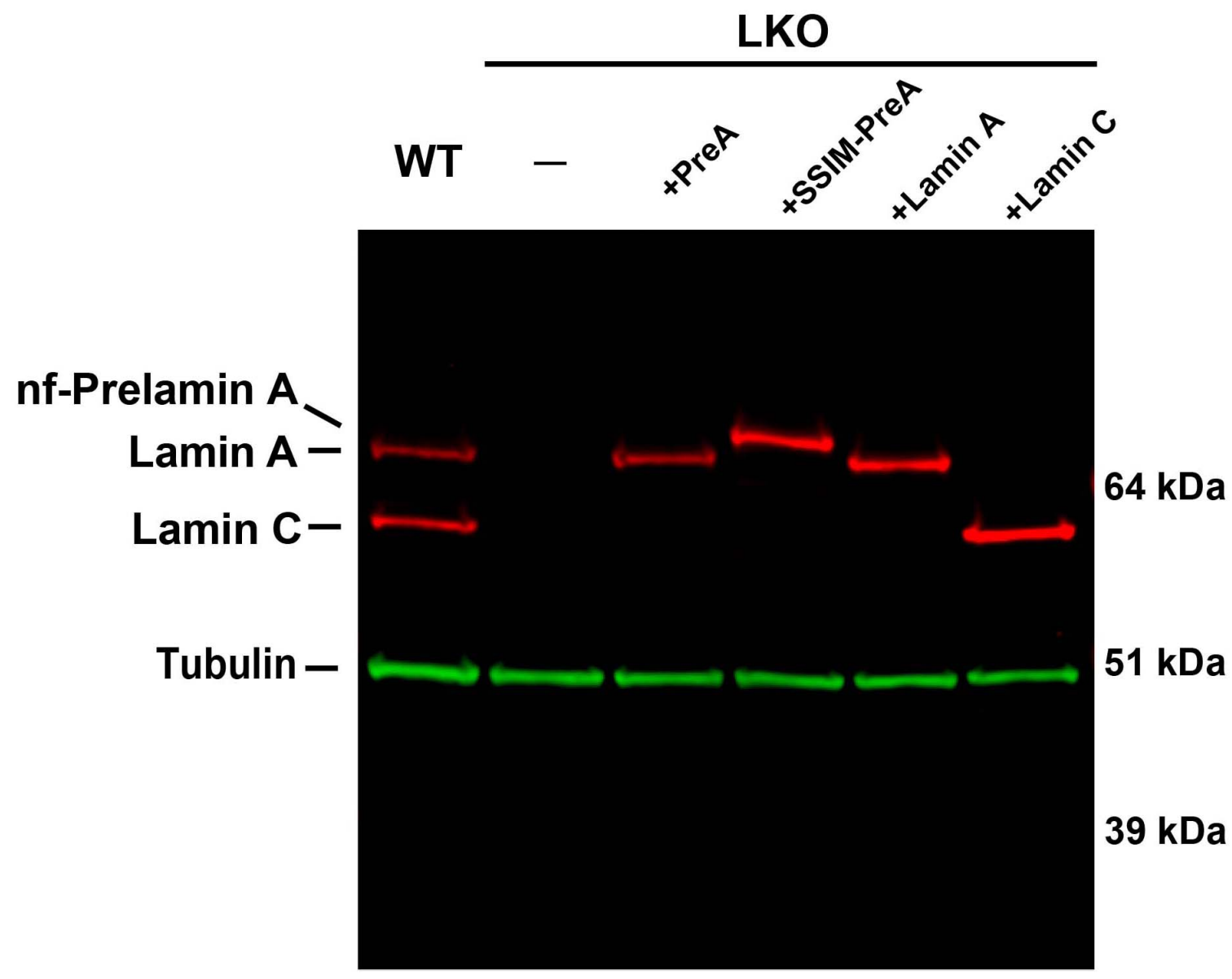

Fig. S4. The analysis of prelamin A, nonfarnesyl-prelamin A, mature lamin A, and lamin C by western blotting. Extracts were prepared from WT cells and LKO cells expressing prelamin A (PreA), SSIM-prelamin A (SSIM-PreA), mature lamin A, or lamin C, and the proteins were fractionated by SDS-PAGE. After transfer to nitrocellulose, the membrane was stained with an antibody against the amino terminus of lamin A/C (*red*) and tubulin (*green*). Molecular weight markers are shown on the right. Note that prelamin A was processed to mature lamin A but the expression of SSIM-prelamin A led to the accumulation of nonfarnesylated prelamin A (nf-Prelamin A).

Journal of Cell Science • Supplementary information

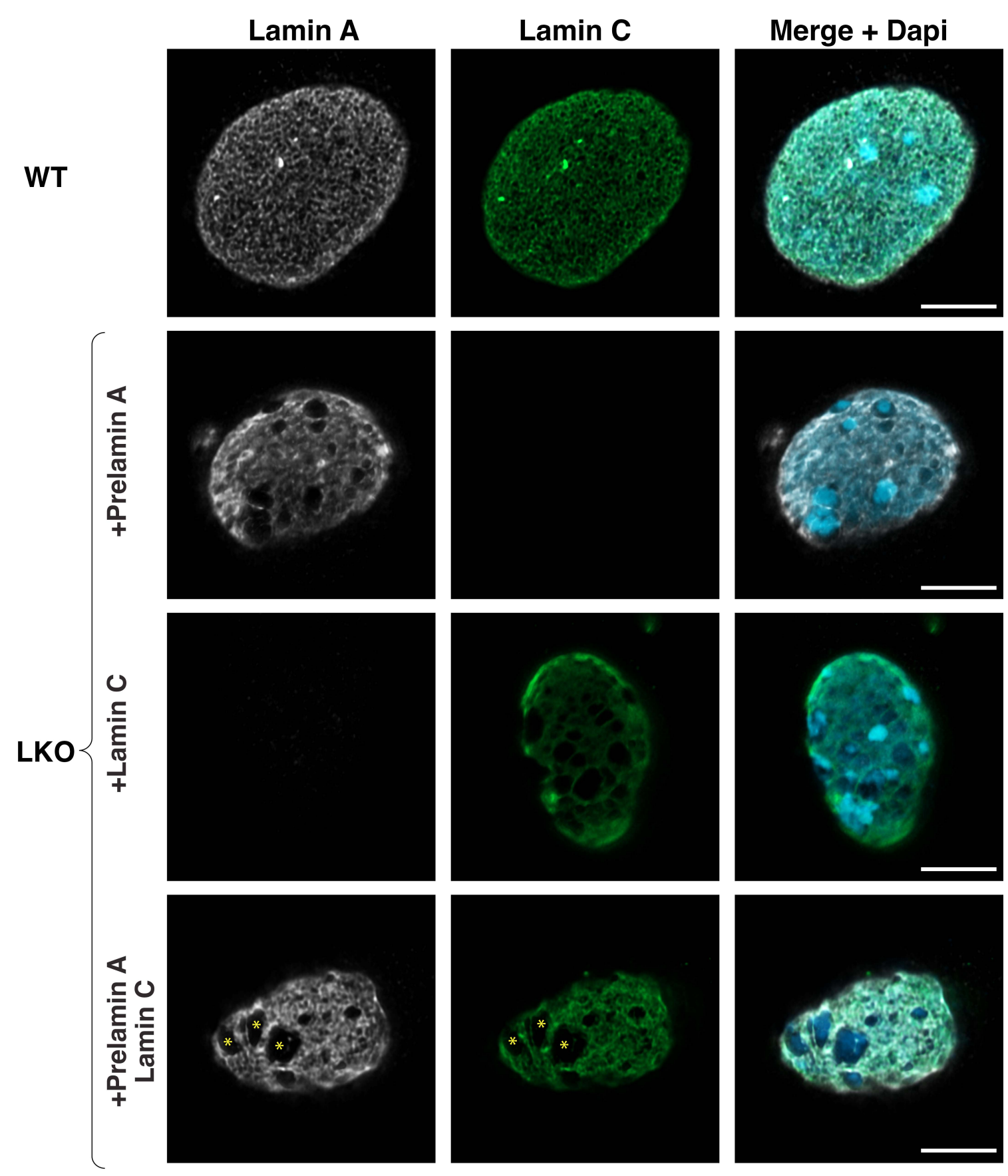

Fig. S5. The expression of lamin A and lamin C together does not form a normal meshwork. LKO cells were transiently transfected with expression vectors for prelamin A and lamin C separately and together. After 48 h, the cells were stained with antibodies against lamin A (*white*) and lamin C (*green*). DNA was stained with Dapi (*blue*). Images were recorded near the top of nuclei. Scale bar, 5 µm. Asterisks mark large holes in the lamin A and lamin C meshworks.

Journal of Cell Science • Supplementary information

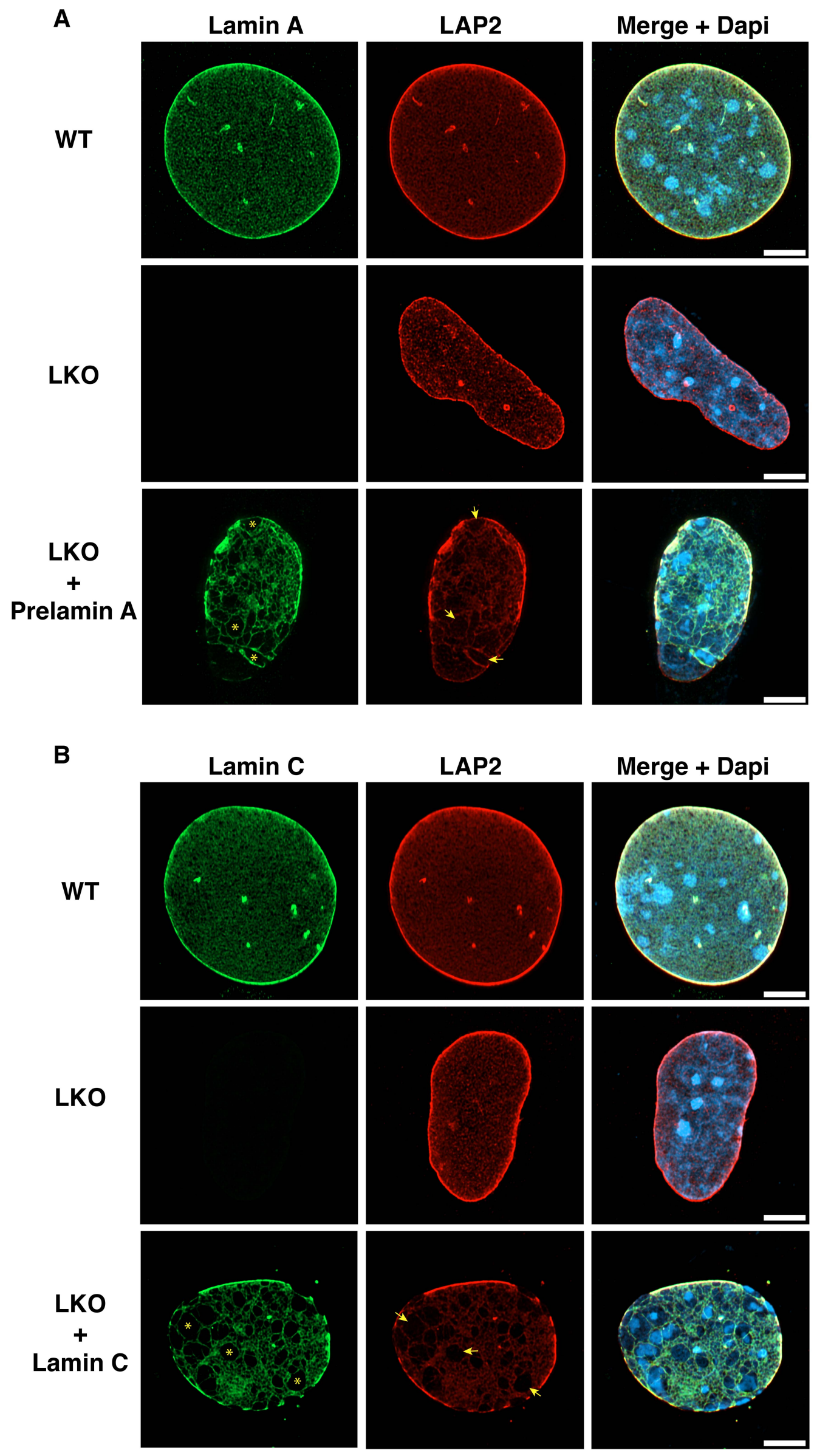

Fig. S6. Lamin A and lamin C expression influence the distribution of LAP2 at the nuclear membrane. LKO cells were transiently transfected with expression vectors for prelamin A or lamin C. After 48 h, the cells were stained with antibodies against (A) lamin A (*green*) and LAP2β (*red*) or (B) lamin C (*green*) and LAP2β (*red*). DNA was stained with Dapi (*blue*). WT and nontransfected LKO cells were included as controls. Images were recorded near the top of nuclei. Scale bar, 5 µm. Asterisks mark large holes in the lamin A and lamin C meshworks. Arrows mark gaps in the distribution of LAP2 that coincide with the large holes in the lamin A and lamin C meshworks.

Journal of Cell Science • Supplementary information

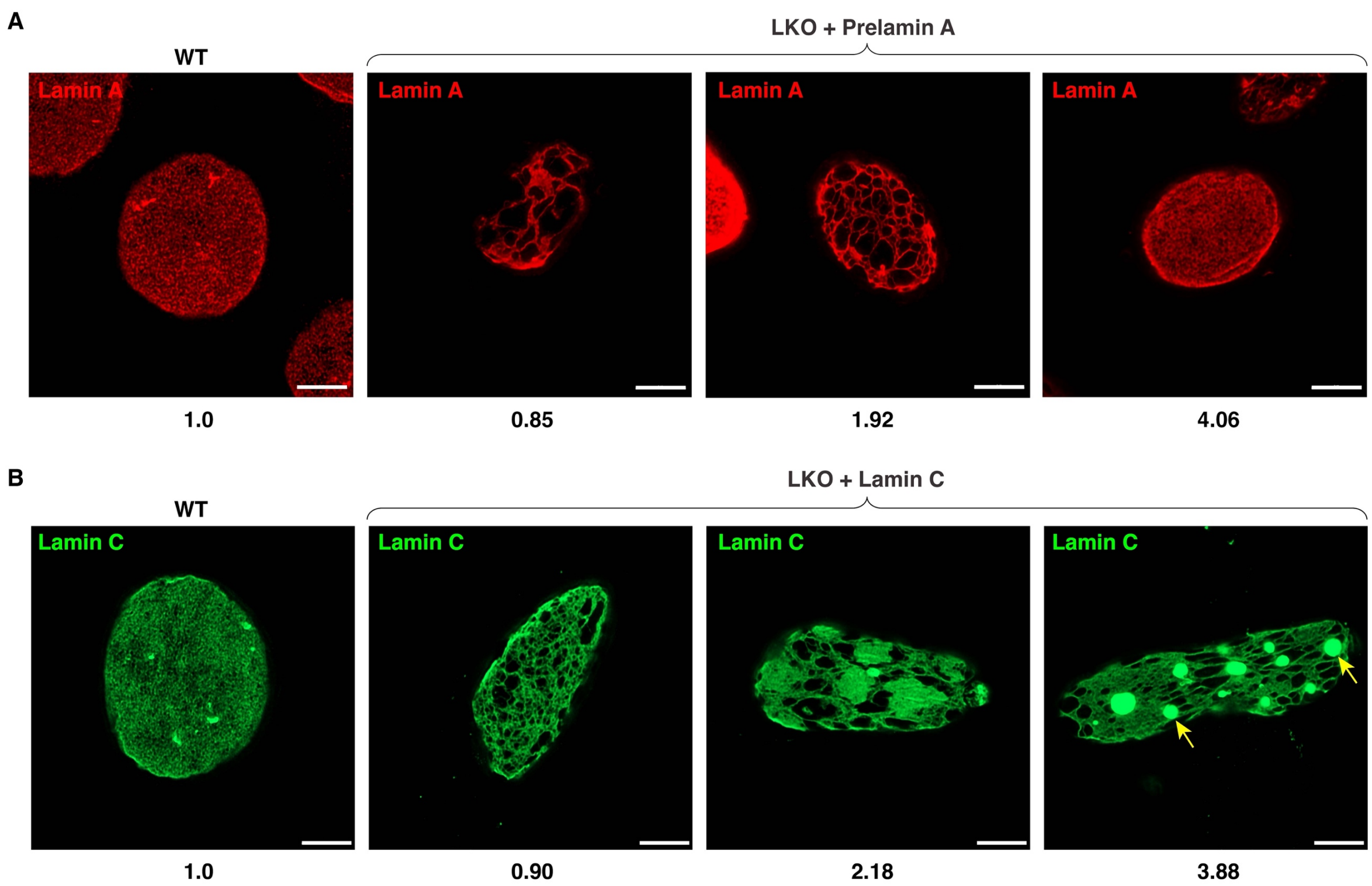

Fig. S7. Expressing lamin A and lamin C at different levels affects their distribution in the nucleus. LKO cells were transfected with plasmids for prelamin A or lamin C and then fixed at 48 h. Wildtype (WT) cells were included as a control. The cells were stained with directly labeled fluorescent antibodies against lamin A and lamin C, and the fluorescence signals were quantified in individual nuclei. Nuclei in LKO cells expressing different levels of nuclear lamin expression (up to fourfold higher than in WT cells) were selected for imaging. To visualize the lamin meshworks, images were recorded near the top of nuclei. The fluorescence signals were adjusted to yield images with similar intensities. (A) Representative images of lamin A (*red*) in WT cells and prelamin A–transfected LKO cells. Scale bar, 5 µm. The levels of lamin A expression are shown beneath each image (with expression in WT cells set at a value of 1.0). (B) Representative microscopy images of lamin C expression (*green*) in WT and lamin C-transfected LKO cells. Scale bar, 5 µm. The levels of lamin C expression are shown beneath each image. Lamin C forms aggregates (arrows) in cells with high levels of lamin C expression.

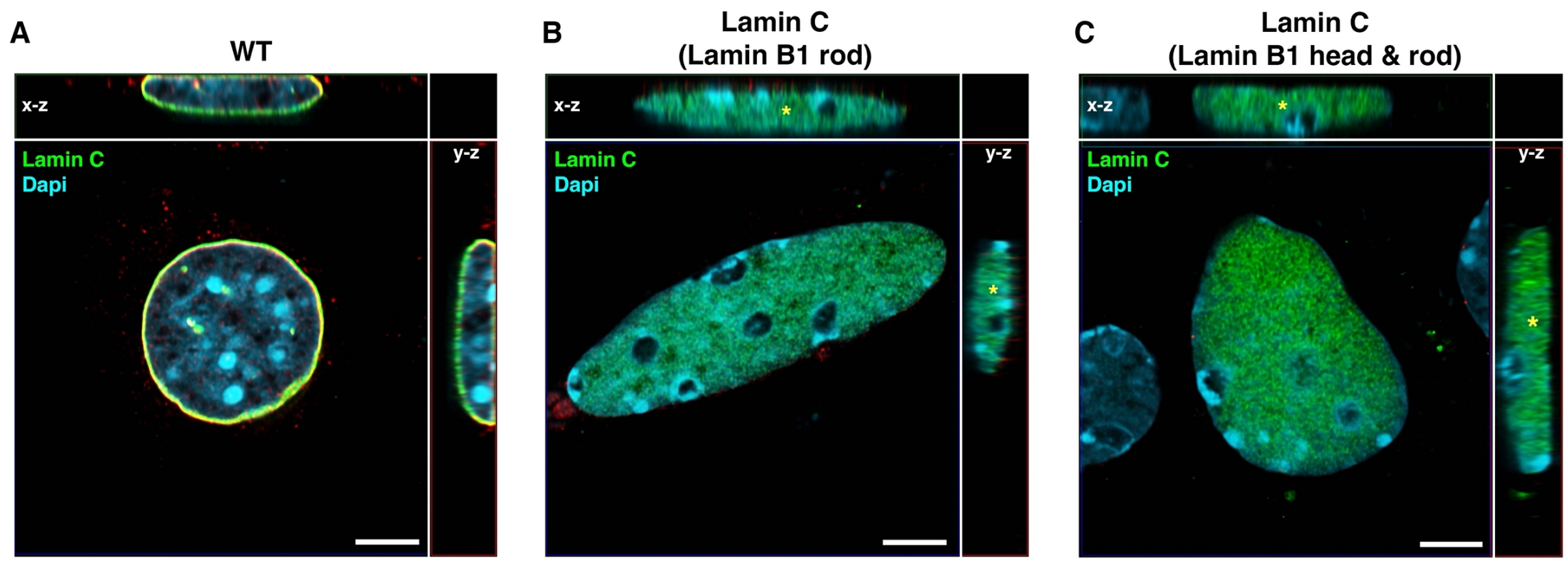

Fig. S8. Replacing lamin C's rod domain with lamin B1's rod domain results in the mislocalization of lamin C into the nucleoplasm. The rod domain (or the rod and head domains) of lamin C were replaced with rod and head domains of lamin B1. Orthogonal images of a single optic section in the *x–z* and *y–z* planes were generated for nuclei in **Figure 6D**. Lamin C (*green*); Dapi (*blue*). Scale bar, 5 µm. Asterisks (*yellow*) identify lamin B1 in the nucleoplasm.

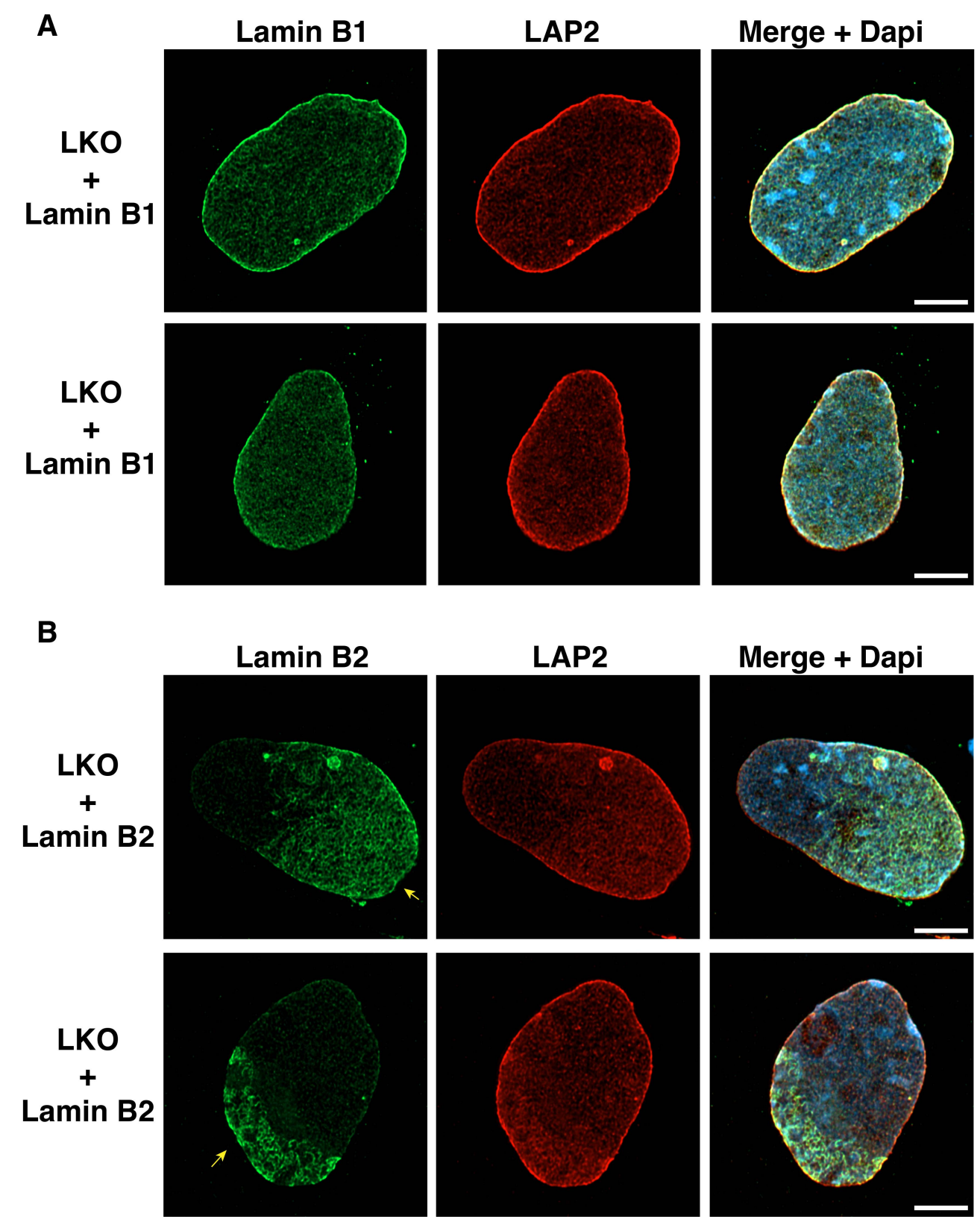

Fig. S9. Lamin B1, but not lamin B2, forms a normal meshwork in LKO cells. LKO cells were transiently transfected with expression plasmids for lamin B1 or lamin B2. After 48 h, the cells were stained with antibodies against (A) lamin B1 (*green*) and LAP2β (*red*) or (B) lamin B2 (*green*) and LAP2β (*red*). DNA was stained with Dapi (*blue*). Images were taken near the top of nuclei. Scale bar, 5 µm. Arrows point to the polarized distribution of lamin B2.

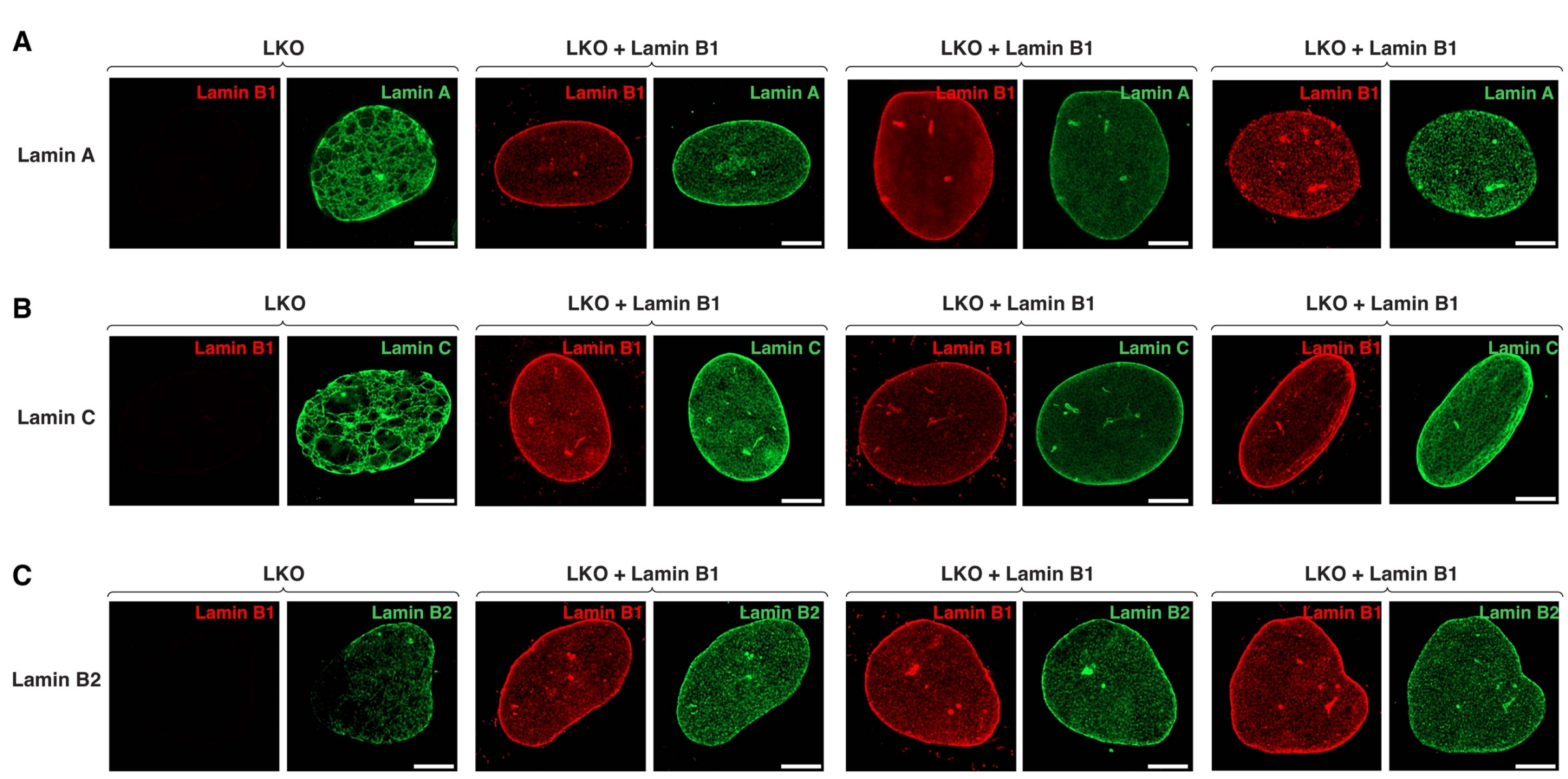

Fig. S10. Lamin B1 rescues the abnormal meshworks formed by lamin A, lamin C, and lamin B2. LKO cells stably expressing a doxycycline (Dox)-inducible lamin B1 construct were transiently transfected with expression plasmids for (A) prelamin A, (B) lamin C, or (C) lamin B2. After 48 h, the cells were stained with antibodies against lamin B1 (*red*) and either lamin A (*green*), lamin C (*green*), or lamin B2 (*green*). Representative images of non-induced LKO cells (LKO) and LKO cells that had been incubated with Dox to induce lamin B1 synthesis (LKO + Lamin B1) are shown. Images were taken near the top of nuclei. Scale bar, 5 µm.

Journal of Cell Science • Supplementary information

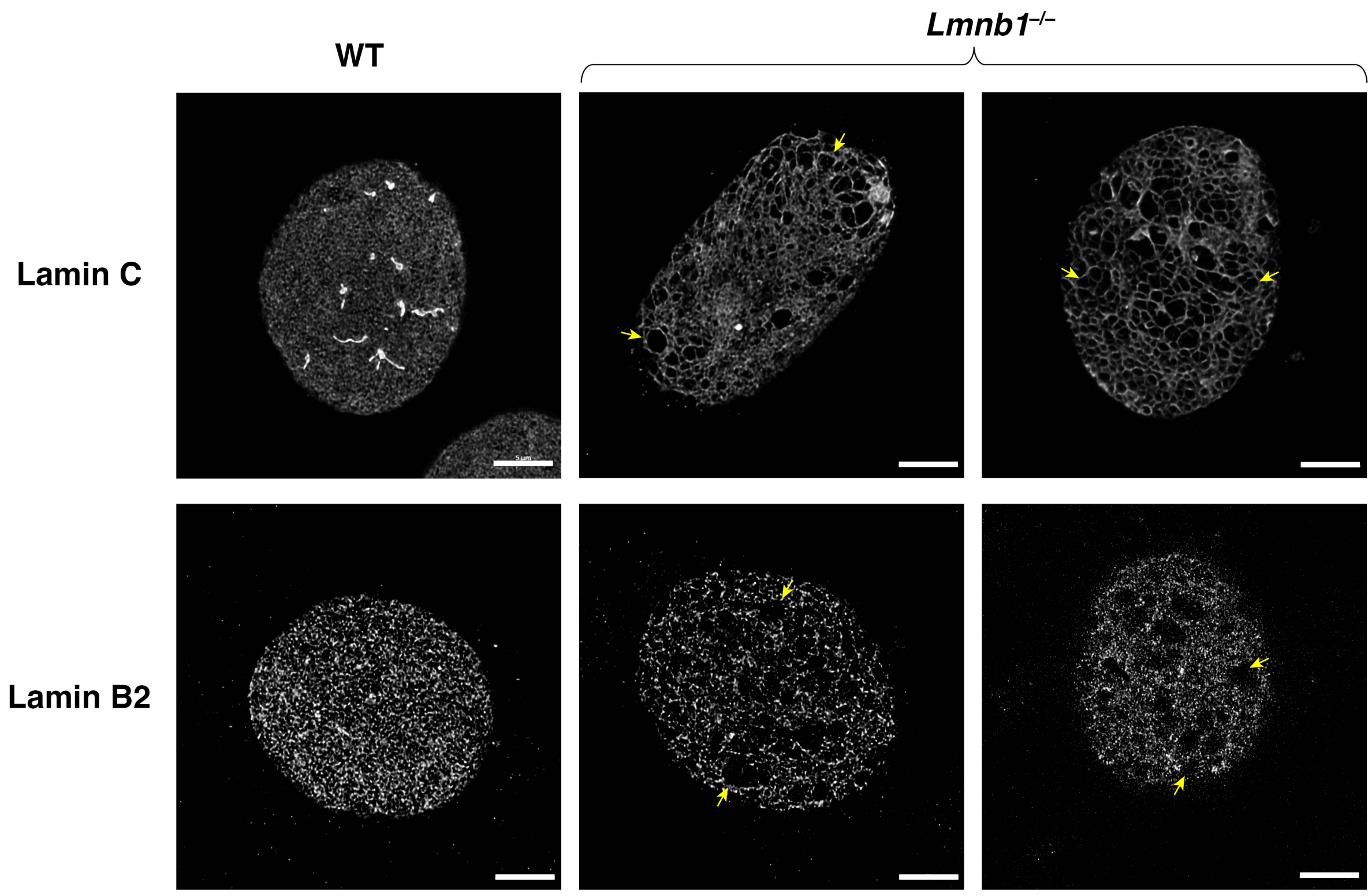

Fig. S11. Lamin C and lamin B2 meshworks are abnormal in *Lmnb1*$^{-/-}$ SMCs. Wild- type (WT) and *Lmnb1*$^{-/-}$ SMCs were stained with antibodies against lamin C or lamin B2. Images were taken near the top of nuclei. Scale bar, 5 µm. Arrows (yellow) point to areas with large gaps in the meshworks.

Full unedited Western blot gels

Suppl. Fig. 1C

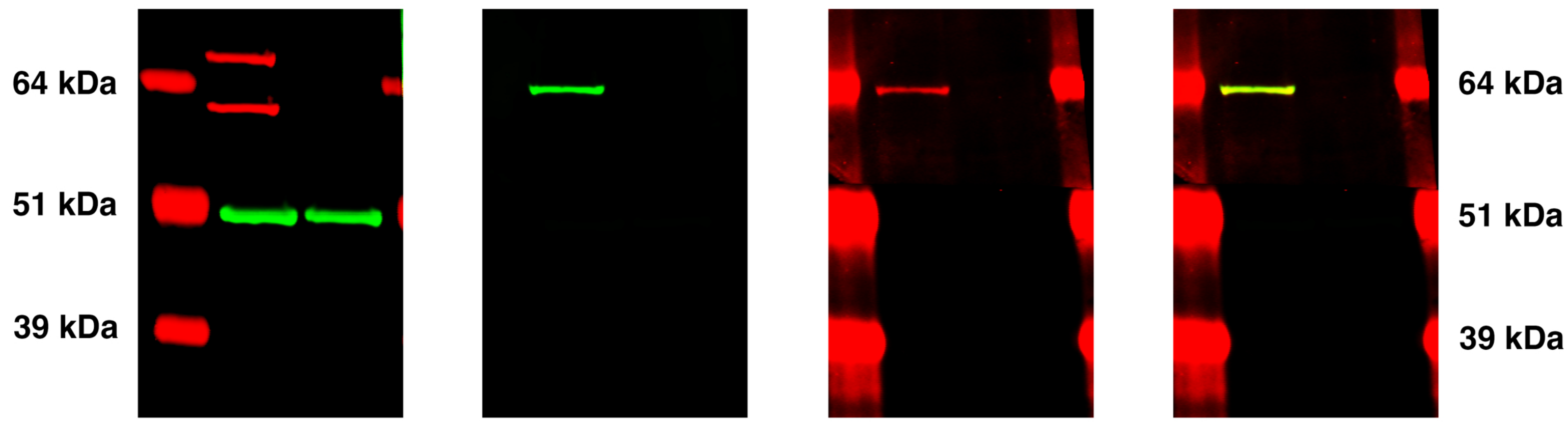

Anti-Lamin A/C (red)
Anti-Tubulin (green)

Anti-Lamin B1 (green)

Anti-Lamin B2 (red)

Anti-Lamin B1 (green)
Anti-Lamin B2 (red)

Suppl. Fig. 4

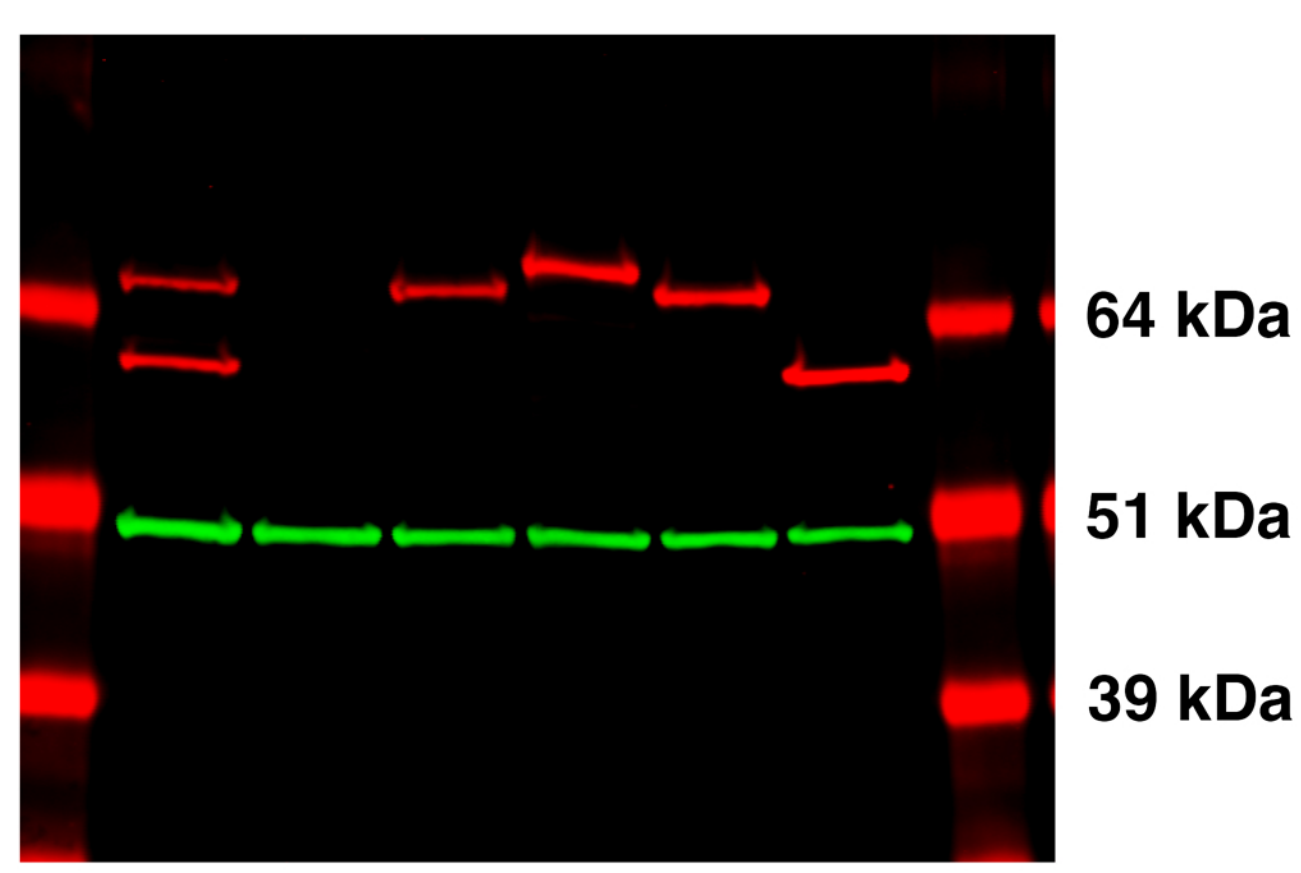

Anti-Lamin A/C (red)
Anti-Tubulin (green)

Fig. 6B

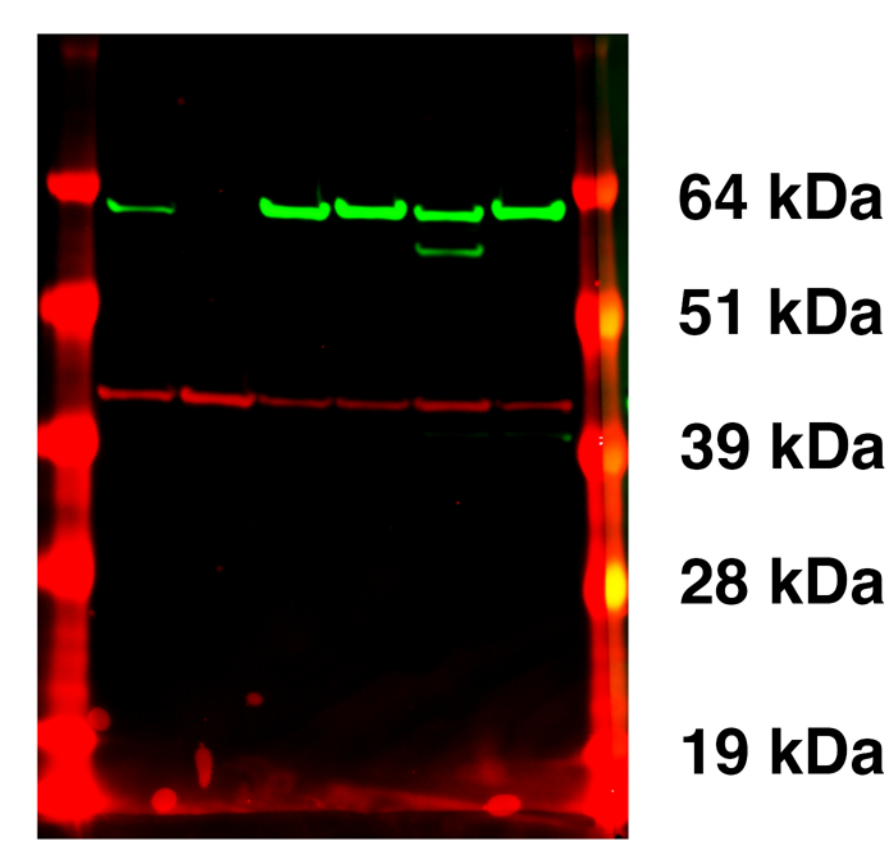

Anti-Lamin C (green)
Anti-Actin (red)

Fig. S12. Blot transparency data.

Journal of Cell Science • Supplementary information

Table S1. Antibodies used for Western blotting and immunocytochemistry.

Antibody Description	Species	Source	Catalog #	Use	Dilution
β-actin	Mouse	Santa Cruz Biotech	SC47778	WB	1:3000
Tubulin	Rat	Novus Bio	NB600-506	WB	1:3000
Lamin A/C	Mouse	Santa Cruz Biotech	SC376248	WB	1:1000
Lamin C	Rabbit	Abcam	Ab314500	WB	1:1500
Lamin B1	Rabbit	Invitrogen	702972	WB	1:1000
Lamin B2	Mouse	ThermoFisher	33-2100	WB	1:500
Lamin A-AF568	Rabbit	Abcam	Ab26300*	IF	1:25
Lamin C-AF488	Rabbit	Abcam	Ab323445	IF	1:200
Lamin B1-AF488	Mouse	Abcam	Ab215324	IF	1:200
Lamin B1-AF568	Mouse	Abcam	Ab215324	IF	1:200
Lamin B2-AF488	Mouse	ThermoFisher	MA3-32100	IF	1:300
Lamin B2-AF555	Mouse	ThermoFisher	MA3-32100	IF	1:300
Nup107	Mouse	ThermoFisher	MA1-10031	IF	1:100
LAP2	Mouse	BD Bioscience	611000	IF	1:2000
LAP2β	Rabbit	Bethyl	A304-841A	IF	1:200
Emerin	Mouse	Fisher Scientific	50-255-2259	IF	1:500
Anti-rabbit IR800	Donkey	LI-COR	926-32213	WB	1:10000
Anti-mouse IR680	Donkey	ThermoFisher	SA5-10170	WB	1:5000
Anti-rat IR800	Donkey	ThermoFisher	SA5-10032	WB	1:5000
Anti-mouse IR680	Donkey	ThermoFisher	SA5-10170	WB	1:5000
Anti-mouse Alexa 488	Donkey	Invitrogen	A21202	IF	1:2000
Anti-rabbit Alexa 488	Donkey	Invitrogen	A21206	IF	1:2000
Anti-mouse Alexa 568	Donkey	Invitrogen	A10037	IF	1:2000
Anti-rabbit Alexa 568	Donkey	Invitrogen	A10042	IF	1:2000
Anti-rabbit Alexa 555+	Donkey	Invitrogen	A32794	IF	1:1000
Anti-mouse Alexa 488+	Donkey	Invitrogen	A32766TR	IF	1:1000

*Labeled with AlexaFluor dye in-house.

Journal of Cell Science • Supplementary information

Table S2. Guide RNAs for lamin gene deletion.

Gene	gRNA 5′ target site	gRNA 3′ target site
Lmna	GGATTGGCCGCTTCTGTGCG	CCAATCGCCGCACCTCTAGA
Lmnb2	CTACAAATTCCGGCGCGCTG	AACGCCGGTCACAAAGAAAA
Lmnb1	GCTGGAACCACTCCACGTGT and GGCGAGAGGCGGGCAGGAGG	TAGTCACCATGTACGCACTC and ATCTTACAACGTGTATGGAT

Table S3. InFusion primers.

Construct	Forward (5′–3′)	Reverse (5′–3′)
Prelamin A	GGGCGGCCGCGAATTATGGAG ACCCCGTCACAGCG	AGGGATGCCACCCGGTTACATGAT GCTGCAGTTCTGG
Mature lamin A	GGGCGGCCGCGAATTATGGAG ACCCCGTCACAG	AGGGATGCCACCCGGGTTAGTAGG AGCGGGTGACTAGGTTGTC
Lamin C	GGGCGGCCGCGAATTATGGAG ACCCCGTCACAGCG	AGGGATGCCACCCGGGTCAGCGGC GGCTGCCACTCA
Lmnb1	GGGCGGCCGCGAATTATGGCG ACCGCGAC	AGGGATGCCACCCGGTCACATAAT GGCACA
Lmnb2	GGGCGGCCGCGAATTATGAGC GCGCCGCATTCG	AGGGATGCCACCCGGTCACATCAG TCGGCAGCCC

Table S4. Mutagenesis primers.

Construct	Forward (5′–3′)	Reverse (5′–3′)
Nf-Lamin B1	TTGAATTTCACATAATGGCACT GCTTTTATTGGATGCTCTTGG	CCAAGAGCATCCAATAAAAGCAG TGCCATTATGTGAAATTCAA
Nf-Lamin B2	GGGCGGCCGCGAATTTAAGCCA CCATGGGGGAGTCGGAATCCA	AGGGATGCCACCCGGTCACATCA GTCGGGAGCCCCTTG
SSIM-prelamin A	CCAGAGCTCCCAGAACAGCAG CATCATGTAACC	GGTTACATGATGCTGCTGTTCTGG GAGCTCTGG

Journal of Cell Science • Supplementary information

Table S5. Lamin C–lamin B1 chimeric protein sequences.

Chimeric construct	Protein sequence
**Lamin B1 head domain–Lamin C rod domain–Lamin C tail domain**	ATATPVQQQRAGSRASAPATPLSPTRLSRLQEKEDLQELNDRL<br>AVYIDRVRSLETENAGLRLRITESEEVVSREVSGIKAAYEAELG<br>DARKTLDSVAKERARLQLELSKVREEFKELKARNTKKEGDLL<br>AAQARLKDLEALLNSKEAALSTALSEKRTLEGELHDLRGQVA<br>KLEAALGEAKKQLQDEMLRRVDAENRLQTLKEELDFQKNIYS<br>EELRETKRRHETRLVEIDNGKQREFESRLADALQELRAQHED<br>QVEQYKKELEKTYSAKLDNARQSAERNSNLVGAAHEELQQS<br>RIRIDSLSAQLSQLQKQLAAKEAKLRDLEDSLARERDTSRRLL<br>AEKEREMAEMRARMQQQLDEYQELLDIKLALDMEIHAYRKL<br>LEGEEERLRLSPSPTSQRSRGRASSHSSQSQGGGSVTKKRKLES<br>SESRSSFSQHARTSGRVAVEEVDEEGKFVRLRNKSNEDQSMG<br>NWQIRRQNGDDPLMTYRFPPKFTLKAGQVVTIWASGAGATH<br>SPPTDLVWKAQNTWGCGSSLRTALINSTGEEVAMRKLVRSLT<br>MVEDNEDDDEDGEELLHHHRVSGSRR*
**Lamin C head domain–Lamin B1 rod domain–Lamin C tail domain**	ETPSQRRATRSGAQASSTPLSPTRITRLQEKEELRELNDRLAVY<br>IDKVRSLETENSALQLQVTEREEVRGRELTGLKALYETELADA<br>RRALDDTARERAKLQIELGKFKAEHDQLLLNYAKKESDLSGA<br>QIKLREYEAALNSKDAALATALGDKKSLEGDLEDLKDQIAQL<br>EASLSAAKKQLADETLLKVDLENRCQSLTEDLEFRKNMYEEEI<br>NETRRKHETRLVEVDSGRQIEYEYKLAQALHEMREQHDAQV<br>RLYKEELEQTYHAKLENARLSSEMNTSTVNSAREELMESRMR<br>IESLSSQLSNLQKESRACLERIQELEDMLAKERDNSRRMLSDR<br>EREMAEIRDQMQQQLSDYEQLLDVKLALDMEISAYRKLLEGE<br>EEEERLSPSPTSQRSRGRASSHSSQSQGGGSVTKKRKLESSESR<br>SSFSQHARTSGRVAVEEVDEEGKFVRLRNKSNEDQSMGNWQI<br>RRQNGDDPLMTYRFPPKFTLKAGQVVTIWASGAGATHSPPTD<br>LVWKAQNTWGCGSSLRTALINSTGEEVAMRKLVRSLTMVED<br>NEDDDEDGEELLHHHRVSGSRR*
**Lamin B1 head domain–Lamin B1 rod domain–Lamin C tail domain**	ATATPVQQQRAGSRASAPATPLSPTRLSRLQEKEELRELNDRL<br>AVYIDKVRSLETENSALQLQVTEREEVRGRELTGLKALYETEL<br>ADARRALDDTARERAKLQIELGKFKAEHDQLLLNYAKKESDL<br>SGAQIKLREYEAALNSKDAALATALGDKKSLEGDLEDLKDQI<br>AQLEASLSAAKKQLADETLLKVDLENRCQSLTEDLEFRKNMY<br>EEEINETRRKHETRLVEVDSGRQIEYEYKLAQALHEMREQHD<br>AQVRLYKEELEQTYHAKLENARLSSEMNTSTVNSAREELMES<br>RMRIESLSSQLSNLQKESRACLERIQELEDMLAKERDNSRRML<br>SDREREMAEIRDQMQQQLSDYEQLLDVKLALDMEISAYRKLL<br>EGEEEEERLRLSPSPTSQRSRGRASSHSSQSQGGGSVTKKRKLE<br>SSESRSSFSQHARTSGRVAVEEVDEEGKFVRLRNKSNEDQSM<br>GNWQIRRQNGDDPLMTYRFPPKFTLKAGQVVTIWASGAGAT<br>HSPPTDLVWKAQNTWGCGSSLRTALINSTGEEVAMRKLVRSL<br>TMVEDNEDDDEDGEELLHHHRVSGSRR*

Journal of Cell Science • Supplementary information

Table S6. Quantitative RT-PCR primers.

Gene or transcript	Forward (5′–3′)	Reverse (5′–3′)
Ppia	TGAGCACTGGAGAGAAAGGA	CCATTATGGCGTGTAAAGTCA
Lmna	CCTATCGAAAGCTGCTGGAG	CCTGAGACTGGGATGAGTGG
Lmnb1	CAACTGACCTCATCTGGAAGAAC	TGAAGACTGTGCTTCTCTGAGC
Lmnb2	GCTGGAGCTGGAGAATGATAG	GTCAGCCAGCTCTGACTCGTA
Lamin C	GTGAGTGGCAGCCGCCGCTGA	TTAATGAAAAGACTTTGGCATGG
Prelamin A	GGTTGAGGACAATGAGGATGA	TGAGCGCAGGTTGTACTCAG
Tmpo	GGAGTGAATCCTGGTCCCATTG	GACTGTTGGAAGAGGAGTAGAGG

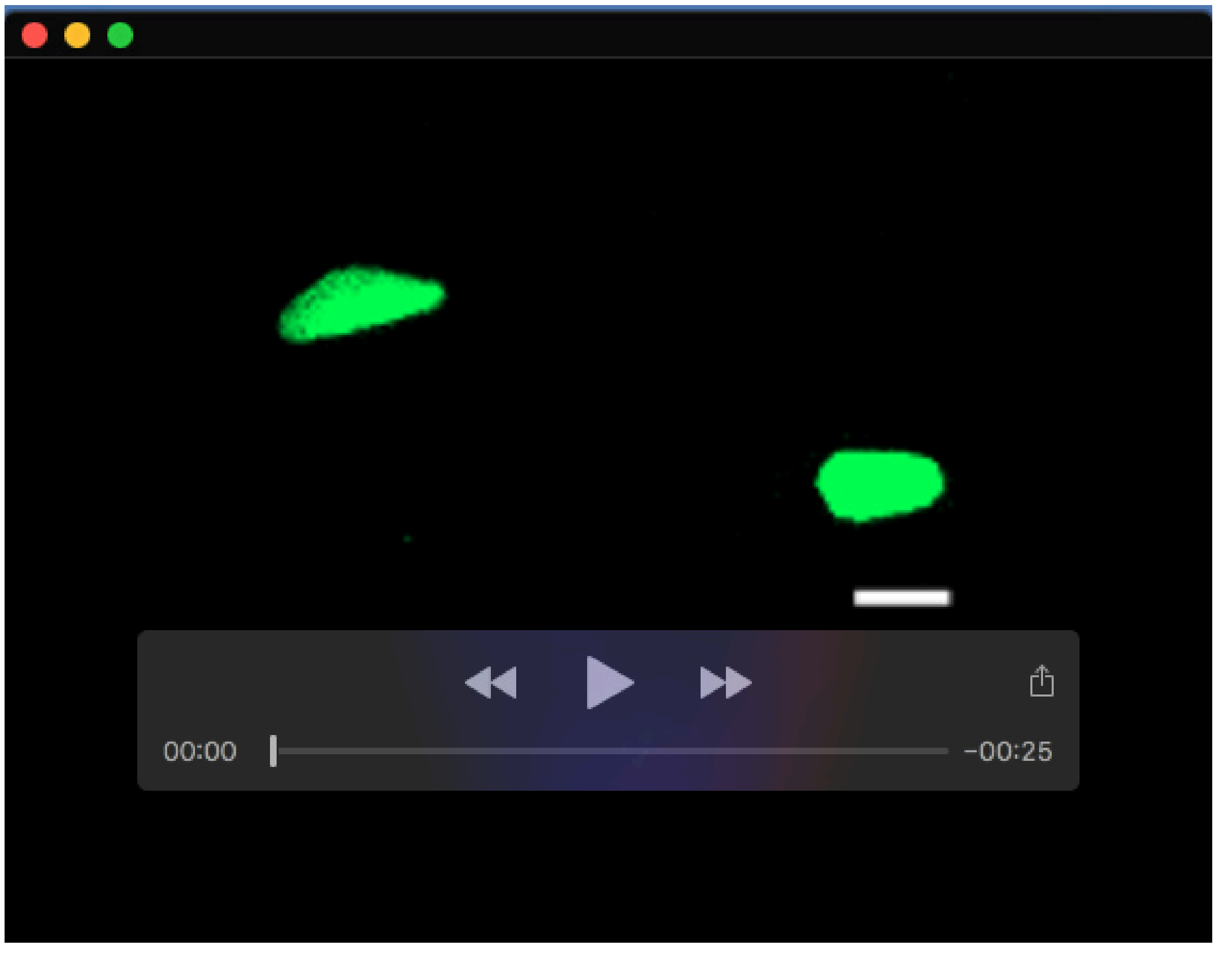

Movie 1. Recording of live LKO cells showing frequent nuclear membrane ruptures. Live LKO cells stably expressing nls-GFP (*green*) were examined by time-lapse confocal fluorescence microscopy at 37°C in the presence of 5% CO_2. Images were collected at a rate of one frame every 10 min for ~9 h. Scale bar, 20 µm. Nuclear membrane ruptures were identified by the appearance of nls-GFP in the cytoplasm. The video display rate is 3 frames/sec. Video stills at six different time points are shown in **Figure 1I**.

Journal of Cell Science • Supplementary information
