# Supplementary material for: Lamin B1 coordinates the assembly of the nuclear lamina
Source: J Cell Sci. 2026 Oct 15;139(19):jcs265156. doi: 10.1242/jcs.265156 (PMC13741955; doi:10.1242/jcs.265156)
Supplement: Supplementary file 2 [file joces-139-265156_review_history.pdf]

Journal of Cell Science

# Lamin B1 coordinates the assembly of the nuclear lamina

JoonYoung R. Kim, Paul H. Kim, Yiping Tu, James Carroll, Jenna P. Koerner, Stephen G. Young and Loren G. Fong

DOI: 10.1242/jcs.265156

**Editor**: Megan King

**Review timeline**
Original submission: 29 June 2026
Editorial decision: 30 July 2026
First revision received: 20 August 2026
Accepted: 5 September 2026

---

**Original submission**

First decision letter

MS ID#: jcs.265156

MS TITLE: Lamin B1 Coordinates the Assembly of the Nuclear Lamin

AUTHORS: Loren G. Fong; Paul H. Kim; JoonYoung R. Kim; Stephen G. Young; Yiping Tu; James Carroll; Jenna P. Koerner

ARTICLE TYPE: Research Article

Dear Loren,

We have now reached a decision on the above manuscript.

As you will see, the reviewers gave quite favorable reports but raised some critical points that will require amendments to your manuscript. I hope that you will be able to carry these out because I would like to be able to accept your paper. Given the enthusiasm from the reviewers and the clarity of the reviews, I expect that I will be able to assess a revised manuscript without the need for further peer review.

Reviewer 1

Kim et al. use a novel model system - smooth muscle cells lacking all mouse nuclear lamin genes in which lamins and variants are expressed - to provide evidence that lamin B1 plays a central role in the assembly of the nuclear lamina. The data are of high quality, the experiments are well-controlled, and the paper is well-written and easily understandable. For this reviewer, the current manuscript is a modern-day version of a Journal of Cell Science classic paper (Gerace et al. J Cell Sci Suppl 1984;1:137-160) that provided some of the earliest insights into the organization and modulation of the nuclear lamina. This new paper will similarly be of interest to a broad readership of cell biologists. I have only a few minor comments the author may want to address.

Supplemental Figure 1B: There is a significant decrease in Tmpo RNA encoding LAP2 in LKO cells compared to WT cells. This should be mentioned in the text. What Tmpo RNA splice variants are being detected? Are the LAP2 protein levels, particularly the predominant inner nuclear membrane isoform LAP2beta, similar in WT and LKO cells? This is relevant as LAP2 localization is examined in several experiments.

© 2026. Published by The Company of Biologists under the terms of the Creative Commons Attribution License (https://creativecommons.org/licenses/by/4.0/).

Lines 53-57: "Each nuclear lamin is a type V intermediate filament protein19 with a conserved structure consisting of a central Î±-helical rod domain flanked by globular head and tail domains. Nuclear lamins assemble into a coiled-coil homodimer that polymerizes head-to-tail into linear strands; those strands then multimerize into higher-order filaments, which are the building blocks of the nuclear lamina20-22." The authors may want to mention that lamins form filaments with a diameter of 3.5 nm in contrast to the canonical 10 nm-diameter cytoplasmic intermediate filaments (Turgay et al. Nature 2017;543:261-264 and their own reference 22).

Lines 64-65: "These modifications [farnesylation] increase the hydrophobicity of the C terminus and are thought to promote lamin B1 and lamin B2 interactions with the inner nuclear membrane." While this is true, integral proteins also play a role B-type lamin interactions with the inner nuclear membrane (see for example, Furukawa et al. EMBO J 1995;14:1626-1636 and Ye Q and Worman HJ. J Biol Chem 1994;269:11306-11311).

Lines 313-134: The author distinguish between LAP1beta and total LAP2: "In nontransfected LKO cells, membrane LAP2 (mainly LAP2beta) is distributed uniformly at the nuclear rim." In Table S2, the authors provide information on both a LAP2 antibody and a LAP2beta antibody. The authors could specify in the figure legends whether they are showing total LAP2 labeling or specifically LAP2beta labeling.

Reviewer 2

SUMMARY OF THE ADVANCE MADE IN THIS PAPER AND ITS POTENTIAL SIGNIFICANCE TO THE FIELD

The type V intermediate filament family in mammals comprises three genes, LMNA, LMNB1, and LMNB2, encoding the A-type and B-type lamins. Previously, detailed dissection of lamin subcellular organization was only pursued in the background of expression of at least some of the lamins. Here, Kim et al. generated a triple lamin knockout smooth muscle cell line, and expressed various lamin transgenes, apparently at physiological levels. This allowed them to examine the subcellular localization and assembly of individual lamins in the absence of any other lamins, and pinpoint molecular requirements for nuclear envelope localization. The ability of individual lamins to form organized fibrillar networks could also be determined, and they were able to identify lamin B1 as a key organizer of lamin organization.

This paper is an interesting and welcome addition to ongoing efforts in the field to systematically dissect molecular contributions of lamin sequence to lamin subcellular localization and function. It clarifies the ability of individual lamins, in the absence of other lamins, to assemble properly and localize to the nuclear envelope. It also sheds new light on the potential hierarchical nature of nuclear lamina assembly.

SUGGESTIONS TO AUTHORS

Major comments

Essential revisions
1. The strategy to flank the coding sequence with gRNA is an important concept that should be conveyed more clearly. In figure 1A, it would be very helpful to graphically depict each lamin gene and the relative location of the guide RNAs on each gene.
2. In multiple figures, the authors show a single image and draw a conclusion regarding an entire population of cells without quantification. I assume that the experiments in KO cells gave clear, unambiguous data and hence there was less need for quantification. Nevertheless, the authors should at minimum report the number of cells examined for each condition. The figures concerned include: Fig 1G, 1H, 1I, Fig 2, Fig 3, Fig 4, Fig 5, Fig 6, Fig 7, Fig 9, Fig 10
3. The main conclusion of the paper, as evident in the title, is that Lamin B1 coordinates the assembly of the nuclear lamina (there is a mistake in the cover page from Editorial Manager: "Lamin B1 Coordinates the Assembly of the Nuclear Lamin"). However, this relies entirely on overexpressing Lamin B1 and other lamins in the LKO cells. While the authors claim to be able to carefully control re-expression of exogenous genes, it may be challenging to completely eliminate potential artefacts from re-expressing LB1. As outlined in the next section, it would then be prudent to

© 2026. Published by The Company of Biologists under the terms of the Creative Commons Attribution License (https://creativecommons.org/licenses/by/4.0/). 

perform the reciprocal experiment of knocking out Lamin B1 and then examining localisation of the other lamins in detail. If this is not feasible, it would be best if the authors acknowledge potential limits to their study.

Potential extensions
1. A key finding in this paper is the role of lamin B1 in organizing the other nuclear lamins. The authors rely on exogenous co-expression of lamin B1 with other nuclear lamins to arrive at this conclusion. To eliminate any uncertainty about control of expression of exogenous transgenes, the authors could consider generating Lmnb1 single knockouts in SMCs, and examine the localization and organization of the other lamins.

Minor comments
Line 84 - 86: "High-resolution confocal microscopy has provided evidence that lamin B1 and the A-type lamins form distinct meshworks at the nuclear rim, each with small, regularly spaced openings"
a. Wouldn't this be better described as high resolution and super-resolution microscopy, and not as confocal microscopy? Reference 35 uses structured illumination, for example, which is not a confocal microscopy method.
b. The authors could consider referencing and incorporating discussion of the findings from: Curr Biol. 2016 Oct 10;26(19):2651-2658 and Proc Natl Acad Sci U S A . 2019 Mar 5;116(10):4307-4315 Both utilize super-resolution microscopy to examine the localization of the nuclear lamins.

---

**First revision**

Author response to reviewers' comments

Our comments are in blue-colored font. Revisions to the manuscript are highlighted in yellow.

**Reviewer #1:**
*Kim et al. use a novel model system - smooth muscle cells lacking all mouse nuclear lamin genes in which lamins and variants are expressed - to provide evidence that lamin B1 plays a central role in the assembly of the nuclear lamina. The data are of high quality, the experiments are well-controlled, and the paper is well-written and easily understandable. For this reviewer, the current manuscript is a modern-day version of a Journal of Cell Science classic paper (Gerace et al. J Cell Sci Suppl 1984;1:137-160) that provided some of the earliest insights into the organization and modulation of the nuclear lamina. This new paper will similarly be of interest to a broad readership of cell biologists. I have only a few minor comments the author may want to address.*

We appreciate Reviewer 1's kind comments and are particularly grateful for the generous comparison of our study to the landmark work by Gerace and colleagues published in *JCS*—high praise indeed. We have addressed Reviewer 1's constructive criticisms in detail below.

1. *Supplemental Figure 1B: There is a significant decrease in Tmpo RNA encoding LAP2 in LKO cells compared to WT cells. This should be mentioned in the text. What Tmpo RNA splice variants are being detected? Are the LAP2 protein levels, particularly the predominant inner nuclear membrane isoform LAP2beta, similar in WT and LKO cells? This is relevant as LAP2 localization is examined in several experiments.*

   The qRT-PCR primers for *Tmpo* were designed to detect all splice variants, revealing reduced expression in LKO cells. As suggested, we have incorporated this finding into the Results section (lines 143–144). Qualitative microscopy also suggested decreased LAP2β protein levels in LKO cells; however, these observations should be interpreted with caution given the non-quantitative nature of the antibody-based detection method (*i.e.*, an unlabeled primary antibody followed by a labeled secondary antibody). Additional studies will be required to rigorously address this issue.

© 2026. Published by The Company of Biologists under the terms of the Creative Commons Attribution License (https://creativecommons.org/licenses/by/4.0/). 

2. *Lines 53-57: "Each nuclear lamin is a type V intermediate filament protein19 with a conserved structure consisting of a central a-helical rod domain flanked by globular head and tail domains. Nuclear lamins assemble into a coiled-coil homodimer that polymerizes head-to-tail into linear strands; those strands then multimerize into higher-order filaments, which are the building blocks of the nuclear lamina20-22." The authors may want to mention that lamins form filaments with a diameter of 3.5 nm in contrast to the canonical 10 nm-diameter cytoplasmic intermediate filaments (Turgay et al. Nature 2017;543:261-264 and their own reference 22).*

   We added that lamins form filaments with a diameter of approximately 3.5-nm (line 61).

3. *Lines 64-65: "These modifications [farnesylation] increase the hydrophobicity of the C terminus and are thought to promote lamin B1 and lamin B2 interactions with the inner nuclear membrane." While this is true, integral proteins also play a role B-type lamin interactions with the inner nuclear membrane (see for example, Furukawa et al. EMBO J 1995;14:1626-1636 and Ye Q and Worman HJ. J Biol Chem 1994;269:11306-11311).*

   We thank the reviewer for bringing this unintended omission to our attention. In the revised manuscript, we have clarified that lamins also interact with integral inner nuclear membrane proteins (lines 71–72).

4. *Lines 313-134: The author distinguish between LAP1beta and total LAP2: "In nontransfected LKO cells, membrane LAP2 (mainly LAP2beta) is distributed uniformly at the nuclear rim." In Table S2, the authors provide information on both a LAP2 antibody and a LAP2beta antibody. The authors could specify in the figure legends whether they are showing total LAP2 labeling or specifically LAP2beta labeling.*

   We agree with the Reviewer that this needs clarification. LAP2 was detected using two antibodies: a pan-LAP2 antibody that recognizes multiple isoforms and an antibody specific for LAP2β. To avoid confusion, we now clarify in the Results section that, unless otherwise indicated, LAP2 detection was performed with the pan-LAP2 antibody (lines 385–388).

**Reviewer #2:**

*The type V intermediate filament family in mammals comprises three genes, LMNA, LMNB1, and LMNB2, encoding the A-type and B-type lamins. Previously, detailed dissection of lamin subcellular organization was only pursued in the background of expression of at least some of the lamins. Here, Kim et al. generated a triple lamin knockout smooth muscle cell line, and expressed various lamin transgenes, apparently at physiological levels. This allowed them to examine the subcellular localization and assembly of individual lamins in the absence of any other lamins, and pinpoint molecular requirements for nuclear envelope localization. The ability of individual lamins to form organized fibrillar networks could also be determined, and they were able to identify lamin B1 as a key organizer of lamin organization.*

*This paper is an interesting and welcome addition to ongoing efforts in the field to systematically dissect molecular contributions of lamin sequence to lamin subcellular localization and function. It clarifies the ability of individual lamins, in the absence of other lamins, to assemble properly and localize to the nuclear envelope. It also sheds new light on the potential hierarchical nature of nuclear lamina assembly.*

We appreciate Reviewer 2’s generous comments on the contributions of our studies “to systematically dissect molecular contributions of lamin sequence to lamin subcellular localization and function” and providing new insights “on the potential hierarchical nature of nuclear lamina assembly.” We have addressed Reviewer 2’s constructive criticisms in detail below.

Major comments:

Essential revisions

© 2026. Published by The Company of Biologists under the terms of the Creative Commons Attribution License (https://creativecommons.org/licenses/by/4.0/).

1. *The strategy to flank the coding sequence with gRNA is an important concept that should be conveyed more clearly. In figure 1A, it would be very helpful to graphically depict each lamin gene and the relative location of the guide RNAs on each gene.*

   We agree with the Reviewer and have added a new graphic showing the locations of the guide RNAs for *Lmna*, *Lmnb1*, and *Lmnb2*. Figure S1A and lines 134–135.

2. *In multiple figures, the authors show a single image and draw a conclusion regarding an entire population of cells without quantification. I assume that the experiments in KO cells gave clear, unambiguous data and hence there was less need for quantification. Nevertheless, the authors should at minimum report the number of cells examined for each condition. The figures concerned include: Fig 1G, 1H, 1I, Fig 2, Fig 3, Fig 4, Fig 5, Fig 6, Fig 7, Fig 9, Fig 10.*

   We understand the Reviewer's comment. Our experimental approach relies on evaluating the organization of the nuclear lamina meshwork in transfected cells. Because lamin expression levels in transfected cells are highly variable, it was essential to selectively analyze cells expressing lamin proteins at wild-type (WT) levels. In each experiment, we first quantified lamin expression levels in WT control cells and then performed high-resolution microscopy only on transfected cells expressing lamin proteins within the WT range. Because fewer than 10% of transfected cells expressed lamin proteins at WT levels, this approach was highly labor-intensive. Nevertheless, we believe that this rigorous selection criterion was responsible for obtaining clear and highly consistent results across independent experiments and different individuals.

   We considered presenting the results as stacked bar graphs showing the proportions of cells with normal versus abnormal meshworks. However, because the phenotype was consistent within each condition, the resulting graphs yielded values of either 0 or 100%. We therefore concluded that representative images provide a more informative presentation of these findings. For Figures 2–10, we examined a minimum of 12–17 cells per condition. This information has now been added to the Results (lines 398–399).

   The microscopy images in Figure 1 show single representative images for emerin, nuclear pore complexes (NPCs), and nuclear membrane ruptures. We presented these findings in this manner because each phenotype has previously been documented in cells lacking full-length nuclear lamins. Nevertheless, to provide additional support for the NPC phenotype, the original manuscript included a low-magnification image in Supplementary Figure 2 showing that NPCs are mislocalized in multiple LKO cells (n > 50 cells). To further support the effect on emerin localization, we now include a low-magnification image in Supplementary Figure 2B showing emerin mislocalization in multiple LKO cells (n > 20 cells).

   Regarding nuclear membrane ruptures, we previously reported that these events occur at very high frequency in cells lacking nuclear lamins (Chen et al., 2018). We therefore respectfully suggest that the representative image in Figure 1I, together with the accompanying video, provides sufficient documentation of this previously established phenotype.

*The main conclusion of the paper, as evident in the title, is that Lamin B1 coordinates the assembly of the nuclear lamina (there is a mistake in the cover page from Editorial Manager: "Lamin B1 Coordinates the Assembly of the Nuclear Lamin"). However, this relies entirely on overexpressing Lamin B1 and other lamins in the LKO cells. While the authors claim to be able to carefully control re-expression of exogenous genes, it may be challenging to completely eliminate potential artefacts from re-expressing LB1. As outlined in the next section, it would then be prudent to perform the reciprocal experiment of knocking out Lamin B1 and then examining localisation of the other lamins in detail. If this is not feasible, it would be best if the authors acknowledge potential limits to their study.*

   We would like to clarify a potential misunderstanding. Our approach (transient transfection of lamins) do not allow the precise control of the re-expression of exogenous genes. Rather, our approach allows us to identify transfected cells that express lamin proteins at WT levels. We recognized from the outset that this would be a significant challenge. In our experience, only ~10% of transfected cells express lamin proteins within the WT range. Also, quantifying lamin

© 2026. Published by The Company of Biologists under the terms of the Creative Commons Attribution License (https://creativecommons.org/licenses/by/4.0/).

expression using conventional immunofluorescence methods—unlabeled primary antibodies followed by labeled secondary antibodies—is susceptible to variability due to antibody amplification. These factors made the experiments technically challenging.

We believe that combining directly labeled lamin antibodies, which eliminate the variable amplification associated with secondary antibodies, with using advanced microscopy software capable of quantitatively measuring fluorescence signals in original Zen microscope images enabled us to reliably identify cells expressing lamins at WT levels. This approach made it feasible to examine the meshworks formed by individual nuclear lamins, as well as the meshworks formed when different lamins were expressed together.

Potential extensions

*A key finding in this paper is the role of lamin B1 in organizing the other nuclear lamins. The authors rely on exogenous co-expression of lamin B1 with other nuclear lamins to arrive at this conclusion. To eliminate any uncertainty about control of expression of exogenous transgenes, the authors could consider generating Lmnb1 single knockouts in SMCs, and examine the localization and organization of the other lamins.*

The successful analysis of cells co-expressing wild-type levels of lamin B1 and one other lamin was made possible by our quantitative approach (described above) and generating a stable cell line carrying a Dox-inducible *Lmnb1* expression construct. By titrating the concentration of Dox, we were able to control lamin B1 expression levels, thereby increasing approximately threefold the likelihood of identifying cells expressing lamin B1 and the other lamin at wild-type levels. Had we instead pursued these questions in *Lmnb1* single-knockout cells, which continue to express multiple lamin proteins, it would not have been possible to determine which lamin was responsible for the observed effects. We therefore reasoned that exogenous co-expression of lamin B1 with each individual nuclear lamin provided the most direct and informative experimental approach.

Nevertheless, we respect the Reviewer’s viewpoint. To further support the importance of lamin B1 in the organization of the lamin meshwork, we examined the lamin C and lamin B2 meshworks in *Lmnb1*$^{-/-}$ SMCs. This is now reported in Suppl. Fig. 11 and described in Results (lines 250–252). As expected, the lamin C and lamin B2 meshworks were abnormal in *Lmnb1*$^{-/-}$ SMCs.

Minor comments

*Line 84 - 86: "High-resolution confocal microscopy has provided evidence that lamin B1 and the A-type lamins form distinct meshworks at the nuclear rim, each with small, regularly spaced openings"*

a. *Wouldn't this be better described as high resolution and super-resolution microscopy, and not as confocal microscopy? Reference 35 uses structured illumination, for example, which is not a confocal microscopy method.*

The Reviewer is right. The description was changed to super-resolution microscopy (lines 93–94).

b. *The authors could consider referencing and incorporating discussion of the findings from: Curr Biol. 2016 Oct 10;26(19):2651-2658 and Proc Natl Acad Sci U S A . 2019 Mar 5;116(10):4307-4315 Both utilize super-resolution microscopy to examine the localization of the nuclear lamins.*

We thank the Reviewer for these helpful suggestions. In the revised manuscript, we have incorporated the super-resolution study by Xie et al. (lines 95 and 97). In the original manuscript, we actually cited the PNAS paper by Nemzi et al. for two principal reasons: first, to highlight their finding that lamin B1 and lamin A/C form distinct meshworks at the nuclear rim (original manuscript, line 85); and second, to note their demonstration that nonfarnesylated lamin B1 is mislocalized to the nucleoplasm (original manuscript, line 67). The

© 2026. Published by The Company of Biologists under the terms of the Creative Commons Attribution License (https://creativecommons.org/licenses/by/4.0/).

citation supporting the first point was inadvertently deleted during editing and has been corrected (line 95).

---

Second decision letter

MS ID#: jcs.265156R1

MS Title: Lamin B1 Coordinates the Assembly of the Nuclear Lamin

Authors: JoonYoung R. Kim; Paul H. Kim; Yiping Tu; James Carroll; Jenna P. Koerner; Stephen G. Young; Loren G. Fong

Article Type: Research Article

Dear Loren,

I am happy to tell you that your manuscript has been accepted for publication in Journal of Cell Science, pending standard publication integrity checks.

© 2026. Published by The Company of Biologists under the terms of the Creative Commons Attribution License (https://creativecommons.org/licenses/by/4.0/).
